# Correction: Prevalence of mental health conditions, sensory impairments and physical disability in people with co-occurring intellectual disabilities and autism compared with other people: a cross-sectional total population study in Scotland

**DOI:** 10.1136/bmjopen-2019-035280corr1

**Published:** 2020-07-09

Dunn K, Rydzewska E, Fleming M, *et al*. Prevalence of mental health conditions, sensory impairments and physical disability in people with co-occurring intellectual disabilities and autism compared with other people: a cross-sectional total population study in Scotland. *BMJ Open* 2020;10:e035280. doi: 10.1136/bmjopen-2019-035280.

This article was previously published with an error. The funding information in the published article was incomplete. The updated funding information is stated below:

This work was supported by the UK Medical Research Council, grant number: MC_PC_17217) and the Scottish Government via the Scottish Learning Disabilities Observatory.

