# Supplementary material for: Prevalence of mental health conditions, sensory impairments and physical disability in people with co-occurring intellectual disabilities and autism compared with other people: a cross-sectional total population study in Scotland
Source: BMJ Open. 2020 Apr 26;10(4):e035280. doi: 10.1136/bmjopen-2019-035280 (PMC7204861; doi:10.1136/bmjopen-2019-035280)
Supplement: Author's manuscript [file bmjopen-2019-035280.draft_revisions.pdf]

BMJ Open is committed to open peer review. As part of this commitment we make the peer review history of every article we publish publicly available.

When an article is published we post the peer reviewers' comments and the authors' responses online. We also post the versions of the paper that were used during peer review. These are the versions that the peer review comments apply to.

The versions of the paper that follow are the versions that were submitted during the peer review process. They are not the versions of record or the final published versions. They should not be cited or distributed as the published version of this manuscript.

BMJ Open is an open access journal and the full, final, typeset and author-corrected version of record of the manuscript is available on our site with no access controls, subscription charges or pay-per-view fees (http://bmjopen.bmj.com).

If you have any questions on BMJ Open's open peer review process please email info.bmjopen@bmj.com

BMJ Open

The prevalence of mental health conditions, sensory impairments, and physical disability in people with co-occurring intellectual disabilities and autism (both conditions together) compared with other people – a total population study of 5,295,403 people

Journal:	*BMJ Open*
Manuscript ID	bmjopen-2019-035280
Article Type:	Original research
Date Submitted by the Author:	28-Oct-2019
Complete List of Authors:	Dunn, Kirsty; University of Glasgow, Health and Wellbeing<br>Rydzewska, Ewelina; University of Glasgow, Institute of Health and Wellbeing<br>Cooper, Sally-Ann; Glasgow University , Institute of Health and Wellbeing
Keywords:	MENTAL HEALTH, Adult psychiatry < PSYCHIATRY, PUBLIC HEALTH

SCHOLARONE™
Manuscripts

The prevalence of mental health conditions, sensory impairments, and physical disability in people with co-occurring intellectual disabilities and autism (both conditions together) compared with other people – a total population study of 5,295,403 people

Kirsty Dunn*
Ewelina Rydzewska (University of Glasgow, Glasgow, UK)
Sally-Ann Cooper (University of Glasgow, Glasgow, UK)

*Corresponding author
Kirsty Dunn
Kirsty.wright@glasgow.ac.uk
07975867751
Institute of Health and Wellbeing
University of Glasgow
Glasgow
UK

Word count: 3,210

Keywords: Intellectual disabilities, autism, mental health conditions, sensory impairments, physical disability.

For peer review only - http://bmjopen.bmj.com/site/about/guidelines.xhtml

Abstract (281 words)

Objectives: To investigate prevalence of mental health conditions, sensory impairments, and physical disability in children, adults, and older adults with co-occurring intellectual disabilities and autism, given its frequent co-occurrence, compared with the general population.

Design: Whole country cohort study.

Setting: General community.

Participants: 5,709 people with co-ocurring intellectual disabilities and autism, compared with 5,289,694 other people.

Outcome measures: Rates and odds ratios (OR) with 95% confidence intervals (95% CI) for mental health conditions, visual impairment, hearing impairment and physical disability in people with co-occurring intellectual disabilities and autism compared with other people, adjusted for age, sex, and interaction between age and co-occurring intellectual disabilities and autism.

Results: All four long-term conditions were markedly more common in children, adults, and older adults with co-occurring intellectual disabilities and autism compared with other people. For mental health, OR=130.8 (95% CI 117.1, 146.1); visual impairment OR=65.9 (95% CI 58.7, 73.9); hearing impairment OR=22.0 (95% CI 19.2, 25.2); physical disability OR=157.5 (95% CI 144.6, 171.7). These ratios are also greater than previously reported for people with *either* intellectual disabilities *or* autism rather than co-occurring intellectual disabilities and autism.

Conclusions: We have quantified the more than double disadvantage for people with co-occurring intellectual disabilities and autism, in terms of additional long-term health conditions. This may well impact on quality of life. It raises challenges for staff working with these people in view of additional

For peer review only - http://bmjopen.bmj.com/site/about/guidelines.xhtml

complexity in assessments, diagnoses, and interventions of additional health conditions, as sensory impairments and mental health conditions in particular, compound with the persons pre-existing communication and cognitive problems in this context. Planning is important, with staff being trained, equipped, resourced and prepared to address the challenge of working for people with these conditions.

Strengths and limitations of this study

- Large scale, whole country study, with a high response rate (94%), so the results are representative of the whole population.
- Intellectual disabilities, autism, and additional long-term conditions were enquired about systematically for everyone in the population.
- The wording of questions was tested in advance, via cognitive question testing during the design of Scotland's Census, 2011.
- Limitations include proxy-reporting.
- People known to have autism/Asperger's syndrome, intellectual disabilities, and the four long-term conditions were reported, rather than each undergoing detailed individual research assessments which are not possible in such large population studies.

For peer review only - http://bmjopen.bmj.com/site/about/guidelines.xhtml

Background

People with intellectual disabilities[1-4] and people with autism[5-7] have more mental and physical health needs than other people. A whole population study of Scotland reported that 21.7% of people with intellectual disabilities also had autism, and 18.0% of people with autism also had intellectual disabilities,[8] so this dually diagnosed group warrant investigation. One would suspect that this population with co-occurring intellectual disabilities and autism is likely to have a high level of additional health needs, but this has received little previous attention. A higher number of additional health needs increases the likelihood of misdiagnosis, and treatment interactions, so requires more complex treatment plans. Hence it is important to investigate long-term additional health needs experienced by people with co-occurring intellectual disabilities and autism.

Some studies have investigated mental ill-health in people with co-occurring intellectual disabilities and autism. A small study of 149 adults with severe or profound intellectual disabilities and autism, living in state-run developmental centres in Louisiana, USA, compared comorbidity with 158 adults with intellectual disabilities without autism in the same centres. The former group had more symptomology for anxiety, mania, schizophrenia, stereotypies, self-injurious behaviour, eating disorders, sexual disorders, and impulse control.[9] A study in Norway compared 62 adults with co-occurring autism and intellectual disabilities under the care of autism services, with 132 adults with intellectual disabilities only receiving intellectual disabilities support.[10] High levels of psychiatric disorders were reported in both groups; 53.2% in the co-occurring intellectual disabilities and autism group, and 17.4% in the intellectual

For peer review only

disabilities only group. An English study of referrals to a specialist intellectual disabilities psychiatric service described 42% of the 137 referred adults who had autism as well as intellectual disabilities to have comorbid psychopathology, most commonly schizophrenia.[11] A study of youth aged 14-20 years age, gender matched 36 people with co-occurring intellectual disabilities and autism with 36 people with intellectual disabilities without autism.[12] They reported the former group to have more episodes of mental ill-health, most commonly depression. A study of people aged 8-29 years with intellectual disabilities and challenging behaviour living in four residential units in England included 69 who also had autism and 13 who did not.[13] They reported a higher prevalence of organic disorders, anxiety and stereotypies in the young people with co-occurring intellectual disabilities and autism. This literature is difficult to summarise overall, as, as well as having small sample sizes, the participants were not drawn from representative populations.

A further study had the advantage of being population-based, but was still small in size.[14] It compared the prevalence, and incidence, of mental ill-health in 77 adults with co-occurring intellectual disabilities and autism with 946 adults with intellectual disabilities without autism, and also with 154 individually age, gender, ability-level, and Down syndrome matched controls. The adults with autism had a higher point prevalence of problem behaviours than the 946 without autism, but compared with the 154 matched controls there was no difference in prevalence, or incidence of either problem behaviours or other mental ill-health.[14] Three large whole population studies have reported that of people with intellectual disabilities, 21.7% reported mental health conditions;[3] and of people with autism, 33.0% of adults,[6] and

For peer review only - http://bmjopen.bmj.com/site/about/guidelines.xhtml

7.6% of children[7] reported mental health conditions, but did not report the rates for people with co-occurring intellectual disabilities and autism.

With regards to sensory impairments, of the 36 matched youth with intellectual disabilities with and without autism, 38.9% with autism reported having visual problems compared with 50.0% without autism, and 13.9% with autism reported having hearing problems compared with 19.4% without autism.[12] An intellectual disabilities register study reported that 95 of the 368 (25.8%) adults with intellectual disabilities who had visual impairment also had markers for autism, compared with 422 of 2,674 (16%) of those who had normal vision, and that 46 of the 60 (76.7%) of the adults with intellectual disabilities and congenital blindness also had markers for autism, compared with only 36 of the 67 (53.7%) with normal vision.[15]

We have not identified other papers on sensory impairments or any on physical disabilities in people with co-occurring intellectual disabilities and autism. However, previous large whole population studies have reported that of people with intellectual disabilities, 12.4% reported blindness/sight loss, 13.1% reported deafness/hearing loss, and 32.6% reported physical disability.[3] Of people with autism, 12.1% of adults[6] and 3.5% of children reported blindness/sight loss,[7] 14.1% of adults[6] and 2.9% of children reported deafness/hearing loss,[,7] and 24.0% of adults[6] and 10.7% of children reported physical disability.[7] They did not, however, report the rates of these conditions for people with co-occurring intellectual disabilities and autism.

Given the frequent overlap of intellectual disabilities and autism, information on the associated comorbid conditions is important, to assist policy makers,

For peer review only - http://bmjopen.bmj.com/site/about/guidelines.xhtml

planners, and practitioners to best adapt services for individuals with co-occurring intellectual disabilities and autism. This paper aims to investigate the prevalence of mental health conditions, sensory impairments, and physical disability in children, adults, and older adults with co-occurring intellectual disabilities and autism, compared with other people.

Methods

Approval

Approval was obtained from the Scottish Government to undertake secondary data analysis of Scotland's Census, 2011.

Data Source

Scotland's Census provides information on Scotland's population every ten years, with the most recent Census on 27th March 2011.[16] The Census provides information on the number and characteristics of Scotland's population and households on the Census date.

It is a legal requirement to complete the census form and households were informed that failure to make a Census return, or supplying false information could result in a £1,000 fine. A very high response rate was achieved, with an estimated 94% of all of Scotland's population completing the Census. The Census team used a Census Coverage Survey with about 40,000 households, to estimate numbers and characteristics of the missing 6%.[17] The Coverage Survey and Census records were deterministically matched to check for duplicates. Individuals estimated to have been missed were then imputed using a subset of characteristics from real individuals. The edit and imputation methodology

For peer review only - http://bmjopen.bmj.com/site/about/guidelines.xhtml
For peer review only

was adapted from the Office for National Statistics rigorous and systematic guidelines.[17]

The Census was completed by the head of each household on behalf of all occupants of the household. We consider it unlikely that people with co-occurring intellectual disabilities and autism completed the form, given the reading age required to do so. Rather, we expect that the people who completed the form on their behalf were parent-carers in family households, support workers for people living in supported accommodation, and the managers/key workers at communal establishments.

Variables

The census included a question to identify people with intellectual disabilities and autism, as well as mental health conditions, sensory impairments, and physical disabilities: 'Do you have any of the following conditions which have lasted, or are expected to last, at least 12 months? Tick all that apply'. There was a choice of ten possible responses to this question: deafness or partial hearing loss; blindness or partial sight loss; learning disability (for example, Down's syndrome); learning difficulty (for example, dyslexia); developmental disorder (for example, Autistic Spectrum Disorder or Asperger's Syndrome); physical disability; mental health condition; long-term illness, disease or condition; other condition. For "other condition" the option of providing more detail in an open text response was provided.

In Scotland, the term "learning disability" is used synonymously with that of "intellectual disabilities" used internationally. Importantly, the Census

For peer review only - http://bmjopen.bmj.com/site/about/guidelines.xhtml

differentiated between intellectual disabilities and specific learning disabilities; and between intellectual disabilities and autism.

During the methodology development for Scotland's Census, 2011, cognitive question testing was undertaken on the questions on long-term health conditions and disabilities. This was to determine whether the questions were answered accurately, and to identify any changes needed to improve data quality and/or the acceptability of the way questions were phrased. Cognitive interviewing is a widely used approach to critically evaluate and improve survey questionnaires.[18] This approach enables researchers to modify survey material to enhance clarity. Retrospective probing was conducted with 102 participants with a variety of sex, age, and health conditions and disabilities (including people with more than one of the conditions). They included people with autism, intellectual disabilities, dyslexia, dyspraxia, speech impairment, mental health conditions (both milder and more serious), and other long-term conditions.[19] Using the cognitive interviewing results, the question aimed to detect autism was improved and rephrased, to better capture this information. The questions on intellectual disability, mental health condition, visual impairment, hearing impairment, and physical disability did not require any modifications.

The Census team imputed answers for the 14.7% who did not tick any of the boxes in question on long-term conditions, based on their free text answers for this question and answers to other health questions in the Census, which increased the completion rate to 97.4%. For the remaining 2.6%, the Census team assumed the most plausible explanation was that the person had no

For peer review only - http://bmjopen.bmj.com/site/about/guidelines.xhtml

long-term condition but did not see the “No condition” check box at the end of the question, and hence recorded them as having no conditions.

Data Analysis

We calculated the number and rate per 1,000 population of children and adults with co-occurring intellectual disabilities and autism. We then calculated the number and percentage of people with mental health conditions, deafness or partial hearing loss, blindness or partial sight loss, and physical disability, for those with co-occurring intellectual disabilities and autism, compared with individuals who do not have co-occurring intellectual disabilities and autism using chi-squared (χ^2) tests. For the whole population we then used logistic regression to calculate the odds ratios (OR: 95% confidence interval, 95% CI) of co-occurring intellectual disabilities and autism statistically predicting the binary response of having each of the four specific types of long-term health conditions, adjusted for age and sex. Sex was binary, with males being the reference group. Age was categorised into groups: 0-15, 16-24, 25-34, 35-44, 45-54, 55-64, 65-74, 75+, with 0-15 years as the reference group. We repeated the regressions, including the interaction term of age x co-occurring intellectual disabilities and autism, as people with the most severe disabilities die earlier, which may affect the profile of additional health problems differently to that seen in the general population. The same reference groups were used. All analysis was conducted using SPSS software version 22.

Patient and Public Involvement

The Scottish Learning Disabilities Observatory, where this research was undertaken, has a specific remit for people with intellectual disabilities and

For peer review only - http://bmjopen.bmj.com/site/about/guidelines.xhtml

people with autism. Its steering group includes partners from third sector organisations and experts by experience, who approved the workplan for this project prior to it commencing. Results from this study will be disseminated for people with intellectual disabilities and autism in an easy-read version via the Scottish Learning Disabilities Observatory website, newsletters, and conference.

Results

Characteristics of the Sample

Scotland's Census, 2011, includes records on 5,295,403 people aged 0-75+ years. 5,709/5,295,403 (1.08/1,000) people had co-occurring intellectual disabilities and autism; of whom 3,769 (66.0%) were male and 1,940 (44.0%) were female. Overall, 2,362/916,331 (2.58/1,000) of the total population of children (0-15 years), and 3,347/4,379,072 (0.76/1,000) adults (16-75+ years) had co-occurring intellectual disabilities and autism.

Compared with the population who did not have co-occurring intellectual disabilities and autism, the population with co-occurring intellectual disabilities and autism had more males (66.0% versus 48.5%; χ^2=703.5; df=1; p<0.001); were younger (χ^2=3894.7; df=7; p<0.001); were more likely to have been born in the UK rather than elsewhere (χ^2=101.9; df=1; p<0.001), revealing lesser geographic mobility; and were no different with regards to Caucasian versus non-Caucasian ethnicity (χ^2=1.1; df=1; p=0.3) (table 1).

- Insert table 1 about here -

For peer review only - http://bmjopen.bmj.com/site/about/guidelines.xhtml

Long-term Health Conditions

Table 2 shows the proportion of people with co-occurring intellectual disabilities and autism, who had each of the four additional long-term health conditions, compared to people who did not have co-occurring intellectual disabilities and autism. Some people in the sample had more than one long-term health condition.

- Insert table 2 about here -

Mental Health Condition

Adjusting for age and sex, given the different distributions compared with the general population, having co-occurring intellectual disabilities and autism had an OR=25.553 (23.933-27.282, 95% CI) in predicting mental health conditions (table 3). When the interaction term was added (age x co-occurring intellectual disabilities and autism), co-occurring intellectual disabilities and autism had an OR=130.803 (117.131-146.070, 95% CI) in predicting a mental health condition (table 3).

- Insert table 3 about here -

Blindness or partial sight loss

Adjusting for age and sex, having co-occurring intellectual disabilities and autism had an OR=36.781 (34.212-39.542, 95% CI) in predicting blindness or partial sight loss (table 4). When the interaction term was added (age x co-occurring intellectual disabilities and autism), co-occurring intellectual disabilities and autism had an OR=65.897 (58.743-73.922, 95% CI) in predicting blindness or partial sight loss (table 4).

For peer review only - http://bmjopen.bmj.com/site/about/guidelines.xhtml

For peer review only

- Insert table 4 about here -

Deafness or partial hearing loss

Adjusting for age and sex, having co-occurring intellectual disabilities and autism had an OR=11.331 (10.430-12.309, 95% CI) in predicting deafness or partial hearing loss (table 5). When the interaction term was added (age x co-occurring intellectual disabilities and autism), co-occurring intellectual disabilities and autism had an OR=21.996 (19.196-25.205, 95% CI) in predicting deafness or partial hearing loss (table 5).

- Insert table 5 about here -

Physical disability

Adjusting for age and sex, having co-occurring intellectual disabilities and autism had an OR=61.159 (57.601-64.938, 95% CI) in predicting physical disability (table 6). When the interaction term was added (age x co-occurring intellectual disabilities and autism), co-occurring intellectual disabilities and autism had an OR=157.535 (144.577-171.655, 95% CI) in predicting physical disability (table 6).

- Insert table 6 about here -

Discussion

Principle findings

For peer review only - http://bmjopen.bmj.com/site/about/guidelines.xhtml

Mental health conditions, blindness or partial sight loss, deafness or partial hearing loss, and physical disability were all significantly more common in people with co-occurring intellectual disabilities and autism than people without these co-occurring conditions. The odds ratios after adjusting for age and sex and the interaction term, were substantial, being 131, 66, 22, and 158 respectively. This is important as each of these conditions are disabling and can significantly impact an individual's quality of life. They contribute to high rates of multi-morbidity, which, on top of communication and cognitive problems due to autism and intellectual disabilities, renders assessments, diagnosis, and treatment of additional health problems more complex than for other people.

Across all age groups, blindness, deafness, and physical disability were more common in females then males with co-occurring intellectual disabilities and autism, unlike the gender ratios in people without co-occurring intellectual disabilities and autism. Mental health conditions were more common in males than females with co-occurring intellectual disabilities and autism, except for the 65+ year group, contrary to the gender ratios in other people. All conditions were more prevalent with increasing age in the people with co-occurring intellectual disabilities and autism, except for physical disability which was more common in the children/youth and older people than in the adults.

Comparison with existing literature

The prevalence of these additional long-term health conditions has seldom been investigated in people with co-occurring intellectual disabilities and autism, particularly in comparison with other people, and never, to our knowledge, as a total population study. All of the long-term health conditions

For peer review only - http://bmjopen.bmj.com/site/about/guidelines.xhtml

For peer review only

were more common than in those without co-occurring intellectual disabilities and autism.

Smaller, less representative studies have reported a higher rate of mental health conditions in adults and youth with co-occurring intellectual disabilities and autism compared with those with intellectual disabilities and without autism,[9-13] but not all.[14] People with autism have been reported to have more mental health conditions than other people (OR=9 in adults and OR=16 in children),[6,7] as have people with intellectual disabilities compared with other people (OR=7),[3] using the same Scotland's Census, 2011 data as in this current paper, whereas the comparable ratio we now report for people with co-occurring intellectual disabilities and autism for mental health conditions was OR=26. Having the co-occurring conditions therefore presents a much higher risk of mental health conditions than either intellectual disabilities or autism on their own.

The previous small study of youth reported lower rates of visual and hearing impairments in those with co-occurring intellectual disabilities and autism (38.9% and 13.9%) compared with those with intellectual disabilities but without autism (50% and 19.4%).[12] This was in contrast with the larger study reporting more autistic symptoms in adults with intellectual disabilities and visual impairments than in adults with intellectual disabilities but without visual impairments.[15] Adults with autism have been reported to have more blindness or partial sight loss, and deafness or partial hearing loss than other people (12.1% and 17.5%),[6] as have children with autism (3.5% and 2.9%),[7] and people (children and adults combined) with intellectual disabilities compared with other people (13.1% and 12.4%),[3] using the same Scotland's Census 2011

For peer review only - http://bmjopen.bmj.com/site/about/guidelines.xhtml

as in this current paper. This current study found the comparable rates for people with co-occurring intellectual disabilities and autism for blindness or partial sight loss, and deafness or partial hearing loss was 21.7% and 19.3% for adults, and 16.6% and 10.3% for children. Having the co-occurring conditions of intellectual disabilities and autism therefore presents a much higher risk of sensory impairments than for children and adults with autism, and for people with intellectual disabilities (although children were not separately studied in the previous report).

Regarding physical disability, 32.6% of people with intellectual disabilities were previously reported to have physical disability.[3] Of people with autism, 24.0% of adults and 10.7% of children reported physical disability.[6,7] These rates are lower than those we report in this current study of people with co-occurring intellectual disabilities and autism – 45.6% of children and 42.2% of adults.

Strengths and limitations

Strengths of the study include its large scale and general population comparison group, whole population coverage and very high response rate so the results are representative of the whole population. Intellectual disabilities, autism, and the long-term health conditions were enquired about systematically for everyone in the population. We believe the results are therefore generalisable to other high-income countries. The cognitive question testing during the design of the Census is a further strength. The Census had clear categories to distinguish between intellectual disabilities, specific learning disability (like dyslexia), and autism.

For peer review only

For peer review only - http://bmjopen.bmj.com/site/about/guidelines.xhtml

Limitations include the proxy-reporting, which may, or may not reflect self-reports. However, without proxy-reports, we would have no information on people unable to self-report due to their disabilities, and a previous review on the topic concluded that overall, proxy reports are a useful addition to determine aspects of well-being in people with intellectual disabilities.[20] Additionally, people were reported who were known to have autism/Asperger's syndrome, intellectual disabilities, and the additional long-term health conditions, rather than detailed individual research assessments being undertaken which are clearly not possible in such large population studies, and may therefore be subject to a degree of error which we were not able to check.

Implications

There is a greater than double disadvantage for people with co-occurring intellectual disabilities and autism, in terms of additional long-term health conditions. We found that, and quantified the extent to which mental health conditions, sensory impairments, and physical disabilities are more common than in people without co-occurring intellectual disabilities and autism, and in people with just intellectual disabilities or just autism. This may well impact on quality of life. It raises challenges for staff working with people with these co-occurring conditions in view of the additional complexity in assessments, diagnoses, and interventions, as sensory impairments and mental health conditions in particular, interact with the persons pre-existing communication and cognitive problems in this context. Therefore, it is important that these co-occurring conditions are planned for with staff being trained, equipped, resourced and prepared to address the challenge.

For peer review only - http://bmjopen.bmj.com/site/about/guidelines.xhtml

References

1. Cooper S-A, McLean G, Guthrie B, McConnachie A, Mercer S, Sullivan F, Morrison. J. Multiple physical and mental health comorbidity in adults with intellectual disabilities: population-based cross-sectional analysis. *BMC Family Practice* 2015; 16: 110.
2. Hughes-McCormack LA, Rydzewska E, Henderson A., Rintoul J, Macintyre C, Cooper S-A. Prevalence and general health status of people with intellectual disabilities in Scotland -a total population study. *J Epidemiol Community Health 2018;* 72: 78–85.
3. Hughes-McCormack LA, Rydzewska E, Henderson A, Rintoul J, Macintyre C, Cooper S-A. Prevalence of mental ill-health and relationship with physical health in a whole country population of 26,349 people with intellectual disabilities, compared with the general population. *Br J Psychiatry Open,* 2017*;* 3: 243-248.
4. Kinnear D, Morrison J, Allan L, Henderson A, Smiley E, & Cooper S-A. Prevalence of physical conditions and multimorbidity in a cohort of adults with intellectual disabilities with and without Down syndrome: cross-sectional study. *BMJ Open* 2018; *8*:e018292.
5. Croen LA, Zerbo O, Qian Y, Massolo ML, Rich S, Sidney S, Kripke C*.* The health status of adults on the autism spectrum. *Autism* 2015; 19:814–823.
6. Rydzewska E, Hughes-McCormack LA, Gillberg C, Henderson A, MacIntyre C, Rintoul J, & Cooper S-A. Prevalence of long-term health conditions in adults with autism: observational study of a whole country population. *BMJ Open* 2018a; 8(8): e023945.
7. Rydzewska E, Hughes-McCormack LA, Gillberg C, Henderson A, MacIntyre C, Rintoul J, Cooper S-A. Prevalence of sensory impairments, physical and

For peer review only - http://bmjopen.bmj.com/site/about/guidelines.xhtml

intellectual disabilities, and mental health in children and young people with self/proxy-reported autism - observational study of a whole country population. *Autism* 2018; 23:1201-9.

8. Dunn K, Rydzewska E, Macintyre C, Rintoul J, & Cooper S-A. The prevalence and general health status of people with intellectual disabilities and autism co-occurring together: a total population study. *J Inellectual Disabilit Res* 2018, 63: 277-285.
9. Cervantes PE, Matson JL. Comorbid symptomology in adults with autism spectrum disorder and intellectual disability. *J Autism Dev Disord* 2015; *45(12)*: 3961-3970.
10. Bakken TL, Helverschou SB, Eilertsen DE, Heggelund T, Myrbakk E, & Martinsen, H. Psychiatric disorders in adolescents and adults with autism and intellectual disability: A representative study in one county in Norway. *Res Dev Disabil* 2010; *31*: 1669-1677.
11. Tsakanikos E, Sturmey P, Costello H, Holt G, Bouras N. Referral trends in mental health services for adults with intellectual disability and autism spectrum disorders. *Autism* 2007;11:9-17.
12. Bradley E, Bolton P. Episodic psychiatric disorders in teenagers with learning disabilities with and without autism. *Br J Psychiatr* 2006; 189:361-6.
13. Hill J, Furniss F. Patterns of emotional and behavioural disturbance associated with autistic traits in young people with severe intellectual disabilities and challenging behaviours. *Res Dev Disabil* 2006; 27:517-28.
14. Melville CA, Cooper S-A, Morrison J, Smiley E, Allan L, Jackson A, Finlayson J, Mantry D. The prevalence and incidence of mental ill-health in adults with autism and intellectual disabilities. *J Autism Dev Disord* 2008; 38:1676-88.

For peer review only

For peer review only - http://bmjopen.bmj.com/site/about/guidelines.xhtml

15. Kiani, R, Bhaumik, S, Tyrer, F, Bankart, J, Miller, H, Cooper, S-A, Brugha, T. The relationship between autism spectrum disorder and visual impairments among adults with intellectual disability. *Autism Res 2019;* 12:1411-1422.
16. National Records of Scotland. Scotland's Census, 2011. Supporting information. *National Records of Scotland*. 2011 http://www.scotlandscensus.gov.uk/supporting-information.
17. Office for National Statistics. Survey Methodology Bulletin No. 69 – September 2011. 2011 https:// webarchive.nationalarchives.gov.uk/20160108193745/http://www. ons.gov.uk/guide-method/method-quality/survey-methodologybulletin/smb-69/index.html.
18. Willis GB. Cognitive interviewing: A tool for improving questionnaire design. Sage Publications; 2004.
19. General Register Office for Scotland. 2011 Census Question Testing- The Health and Disability Questions (2009). 2009 https://www.scotlandscensus.gov.uk/documents/research/2011-census-healthdisability-questions.pdf.
20. Perkins EA, Berkman KA. Into the unknown: Aging with autism spectrum disorders. *Am J Intellect Dev Disabil* 2012; 117:478-96.

For peer review only

Funding

This work was supported by the UK Medical Research Council, grant number: MC_PC_1717), and the Scottish Government via the Scottish Learning Disabilities Observatory.

For peer review only - http://bmjopen.bmj.com/site/about/guidelines.xhtml

The study sponsor and funders had no role in the study design; in the collection, analysis, and interpretation of data; in the writing of the report; and in the decision to submit the article for publication.

The researchers are independent from the funders.

Competing interests

The authors declare no competing interests.

Author's contributions

KD analysed and interpreted the data, and wrote the first draft of the manuscript. ER contributed to data access, data interpretation, and drafting the manuscript. S-AC conceived and managed the project, interpreted data, and contributed to writing the manuscript. All approved the final version of the manuscript. S-AC is the study guarantor.

Data sharing

Data is available via National Records of Scotland, following project approval.

Acknowledgments

We thank the National Records of Scotland Census team, and the Scottish population who completed the Census.

For peer review only - http://bmjopen.bmj.com/site/about/guidelines.xhtml

Table 1: Characteristics of people with and without, co-occurring intellectual disabilities and autism

	**People without co-occurring intellectual disabilities and autism**<br>N=5,289,694 (100%)<br>Number (%)	**People with co-occurring intellectual disabilities and autism**<br>N=5,709 (100%)<br>Number (%)
**Gender***		
Males	2,563,675 (48.5%)	3,769 (66.0%)
Females	2,726,019 (51.5%)	1,940 (44.0%)
**Age groups***		
0-15	913,969 (17.3%)	2,362 (41.4%)
16-24	631,094 (11.9%)	1,394 (24.4%)
25-34	666,725 (12.6%)	602 (10.5%)
35-44	734,304 (13.9%)	450 (7.9%)
45-54	786,355 (14.9%)	401 (7.0%)
55-64	667,157 (12.6%)	256 (4.5%)
65+	890,090 (16.8%)	244 (4.3%)
**Country of birth***		
UK	4,920,614 (93.0%)	5,505 (96.4%)
Other Europe	172,160 (3.3%)	83 (1.5%)
Africa	46,708 (0.9%)	34 (0.6%)
Middle East and Asia	104,480 (2.0%)	50 (0.9%)
The Americas and the Caribbean	33,325 (0.6%)	28 (0.5%)
Other	12,407 (0.2%)	9 (0.2%)
**Ethnicity**		
White	5,078,910 (96.0%)	5,497 (96.3%)
Asian	140,542 (2.7%)	136 (2.4%)
Mixed/multiple ethnicities	19,775 (0.4%)	40 (0.7%)

For peer review only - http://bmjopen.bmj.com/site/about/guidelines.xhtml

African	29,615 (0.6%)	23 (0.4%)
Caribbean or black	6,536 (0.1%)	4 (0.1%)
Other ethnic groups	14,316 (0.3%)	9 (0.2%)

*People with co-occurring intellectual disabilities and autism versus people without co-occurring intellectual disabilities and autism; p<0.01

For peer review only

For peer review only - http://bmjopen.bmj.com/site/about/guidelines.xhtml

Table 2: Prevalence of comorbidities in people with and without co-occurring intellectual disabilities and autism by age and sex

People with co-occurring intellectual disabilities and autism									
**Condition**	**Children/youth, 0-15 years** N=2,362			**Adults, 16-64 years** N=3,103			**Older people, 65+ years** N=244		
	**M** N=1,563 (100%)	**F** N=799 (100%)	**Total** N=2,362 (100%)	**M** N= 2,073 (100%)	**F** N= 1,030 (100%)	**Total** N= 3,103 (100%)	**M** N= 133 (100%)	**F** N= 111 (100%)	**Total** N= 244 (100%)
**Mental health condition**	328 (21.0%)	152 (19.0%)	480 (20.3%)	768 (37.0%)	377 (36.6%)	1145 (36.9%)	80 (60.2%)	80 (72.1%)	160 (65.6%)
**Blindness/partial sight loss**	214 (13.7%)	177 (22.2%)	391 (16.6%)	355 (17.1%)	220 (21.4%)	575 (18.5%)	71 (53.3%)	80 (72.1%)	151 (61.9%)
**Deafness/partial hearing loss**	148 (9.5%)	95 (11.9%)	243 (10.3%)	301 (14.5%)	190 (18.4%)	491 (15.8%)	73 (54.9%)	81 (73.0%)	154 (63.1%)
**Physical disability**	618 (39.5%)	458 (57.3%)	1,076 (45.6%)	719 (34.7%)	508 (49.3%)	1,227 (39.5%)	86 (64.7%)	99 (89.2%)	185 (75.8%)
**People without co-occurring intellectual disabilities and autism**									
**Condition**	**Children/youth, 0-15 years** N=913,969			**Adults, 16-64 years** N=3,485,635			**Older people, 65+ years** N=890,090		
	**M** N=467,543 (100%)	**F** N=446,426 (100%)	**Total** N= 913,969 (100%)	**M** N= 1,712,526 (100%)	**F** N= 1,773,109 (100%)	**Total** N= 3,485,635 (100%)	**M** N= 383,606 (100%)	**F** N= 506,484 (100%)	**Total** N= 890,090 (100%)
**Mental health condition**	1,861 (0.4%)	980 (0.2%)	2,841 (0.3%)	92,308 (5.4%)	95,108 (5.4%)	187,416 (5.4%)	14,760 (3.8%)	26,141 (5.2%)	40,901 (4.6%)
**Blindness/partial sight loss**	1,793 (0.4%)	1,439 (0.3%)	3,232 (0.4%)	24,129 (1.4%)	16,954 (1.0%)	41,083 (1.2%)	30,389 (7.9%)	49,839 (9.8%)	80,228 (9.0%)
**Deafness/partial hearing loss**	2,731 (0.6%)	2,225 (0.5%)	4,956 (0.5%)	70,543 (4.1%)	48,727 (2.7%)	119,270 (3.4%)	111,447 (29.1%)	114,393 (22.6%)	225,840 (25.4%)
**Physical disability**	3,637 (0.8%)	2,799 (0.6%)	6,436 (0.7%)	81,655 (4.8%)	82,968 (4.7%)	164,623 (4.7%)	73,759 (19.2%)	109,103 (21.5%)	182,862 (20.5%)

For peer review only - http://bmjopen.bmj.com/site/about/guidelines.xhtml

Table 3: Independent predictors of mental health conditions in the whole population

Characteristic		Regression 1		Regression 2 (including the interaction term: age x co-occurring intellectual disabilities and autism)	
		**Odds ratio**	**95% confidence interval**	**Odds ratio**	**95% confidence interval**
Co-occurring intellectual disabilities and autism	Not present (reference)	-	-	-	-
	Co-occurring intellectual disabilities and autism	25.553	23.933-27.282	130.803	117.131-146.070
Gender	Male (reference)	-	-	-	-
	Female	1.275	1.264-1.286	1.275	1.264-1.286
Sex	0-15 (reference)	-	-	-	-
	16-24	9.449	9.039-9.877	11.322	10.781-11.890
	25-34	21.168	20.285-22.090	25.305	24.134-26.532
	35-44	29.725	28.497-31.004	35.493	33.866-37.199
	45-54	29.186	27.983-30.441	34.835	33.239-36.507
	55-64	22.633	21.633-23.617	26.997	25.750-28.304
	65+	19.319	18.518-20.155	23.005	21.947-24.115
Age x both intellectual disabilities and autism	0-15 (reference)	-	-	-	-
	16-24	-	-	0.152	0.129-0.178
	25-34	-	-	0.101	0.083-0.123
	35-44	-	-	0.083	0.067-0.104
	45-54	-	-	0.089	0.071-0.112
	55-64	-	-	0.118	0.090-0.154
	65+	-	-	0.308	0.238-0.423
Constant	-	0.002	-	0.002	-

For peer review only - http://bmjopen.bmj.com/site/about/guidelines.xhtml

Table 4: Independent predictors of blindness/partial sight loss in the whole population

Characteristic		Regression 1		Regression 2 (including the interaction term: age x co-occurring intellectual disabilities and autism)	
		**Odds ratio**	**95% confidence interval**	**Odds ratio**	**95% confidence interval**
Co-occurring intellectual disabilities and autism	Not present (reference)	-	-	-	-
	Co-occurring intellectual disabilities and autism	36.781	34.212-39.542	65.897	58.743-73.922
Sex	Male (reference)	-	-	-	-
	Female	1.011	0.999-1.022	1.011	0.999-1.022
Age	0-15 (reference)	-	-	-	-
	16-24	1.557	1.482-1.636	1.663	1.579-1.751
	25-34	1.824	1.739-1.913	1.914	1.821-2.011
	35-44	2.548	2.439-2.663	2.687	2.567-2.813
	45-54	4.416	4.241-4.599	4.668	4.475-4.870
	55-64	7.503	7.215-7.803	7.928	7.609-8.260
	65+	31.064	29.947-32.222	32.750	31.512-34.036
Age x both intellectual disabilities and autism	0-15 (reference)	-	-	-	-
	16-24	-	-	0.433	0.354-0.528
	25-34	-	-	0.669	0.521-0.844
	35-44	-	-	0.529	0.411-0.681
	45-54	-	-	0.368	0.286-0.474
	55-64	-	-	0.278	0.208-0.372
	65+	-	-	0.250	0.188-0.331
Constant	-	0.003	-	0.003	-

For peer review only - http://bmjopen.bmj.com/site/about/guidelines.xhtml

Table 5: Independent predictors of deafness/partial hearing loss in the whole population

Characteristic		Regression 1		Regression 2 (including the interaction term: age x co-occurring intellectual disabilities and autism)	
		**Odds ratio**	**95% confidence interval**	**Odds ratio**	**95% confidence interval**
Co-occurring intellectual disabilities and autism	Not present (reference)	-	-	-	-
	Co-occurring intellectual disabilities and autism	11.331	10.430-12.309	21.996	19.196-25.205
Gender	Male (reference)	-	-	-	-
	Female	0.687	0.682-0.693	0.688	0.682-0.693
Sex	0-15 (reference)	-	-	-	-
	16-24	1.557	1.496-1.621	1.588	1.524-1.654
	25-34	2.358	2.274-2.446	2.408	2.321-2.499
	35-44	4.237	4.099-4.379	4.347	4.203-4.495
	45-54	8.546	8.285-8.815	8.769	8.496-9.051
	55-64	18.761	18.204-19.336	19.243	18.659-19.845
	65+	69.646	67.633-71.719	71.378	69.269-73.552
Age x both intellectual disabilities and autism	0-15 (reference)	-	-	-	-
	16-24	-	-	0.633	0.507-0.790
	25-34	-	-	0.735	0.569-0.948
	35-44	-	-	0.394	0.296-0.523
	45-54	-	-	0.288	0.219-0.378
	55-64	-	-	0.215	0.159-0.289
	65+	-	-	0.222	0.165-0.298
Constant	-	0.006	-	0.006	-

For peer review only - http://bmjopen.bmj.com/site/about/guidelines.xhtml

Table 6: Independent predictors of physical disability in the whole population

Characteristic		Regression 1		Regression 2 (including the interaction term: age x co-occurring intellectual disabilities and autism)	
		**Odds ratio**	**95% confidence interval**	**Odds ratio**	**95% confidence interval**
Co-occurring intellectual disabilities and autism	Not present (reference)	-	-	-	-
	Co-occurring intellectual disabilities and autism	61.159	57.601-64.938	157.535	144.577-171.655
Gender	Male (reference)	-	-	-	-
	Female	1.063	1.055-1.070	1.063	1.055-1.070
Sex	0-15 (reference)	-	-	-	-
	16-24	1.440	1.388-1.495	1.573	1.513-1.637
	25-34	2.603	2.519-2.690	2.857	2.760-2.958
	35-44	5.869	5.699-6.043	6.465	6.267-6.670
	45-54	10.606	10.312-10.908	11.662	11.318-12.017
	55-64	20.730	20.166-21.310	22.756	22.096-23.436
	65+	43.680	42.517-44.875	47.894	46.530-49.298
Age x both intellectual disabilities and autism	0-15 (reference)	-	-	-	-
	16-24	-	-	0.410	0.355-0.472
	25-34	-	-	0.344	0.287-0.414
	35-44	-	-	0.123	0.100-0.152
	45-54	-	-	0.075	0.061-0.094
	55-64	-	-	0.044	0.034-0.057
	65+	-	-	0.050	0.039-0.066
Constant	-	0.006	-	0.005	-

For peer review only - http://bmjopen.bmj.com/site/about/guidelines.xhtml

BMJ Open

The prevalence of mental health conditions, sensory impairments, and physical disability in people with co-occurring intellectual disabilities and autism compared with other people – a cross-sectional total population study of 5,295,403 people in Scotland

Journal:	*BMJ Open*
Manuscript ID	bmjopen-2019-035280.R1
Article Type:	Original research
Date Submitted by the Author:	23-Jan-2020
Complete List of Authors:	Dunn, Kirsty; University of Glasgow, Health and Wellbeing<br>Rydzewska, Ewelina; University of Glasgow, Institute of Health and Wellbeing<br>Fleming, Michael ; University of Glasgow, Public Health<br>Cooper, Sally-Ann; Glasgow University , Institute of Health and Wellbeing
**Primary Subject Heading**:	Mental health
Secondary Subject Heading:	Public health
Keywords:	MENTAL HEALTH, Adult psychiatry < PSYCHIATRY, PUBLIC HEALTH

SCHOLARONE™
Manuscripts

I, the Submitting Author has the right to grant and does grant on behalf of all authors of the Work (as defined in the below author licence), an exclusive licence and/or a non-exclusive licence for contributions from authors who are: i) UK Crown employees; ii) where BMJ has agreed a CC-BY licence shall apply, and/or iii) in accordance with the terms applicable for US Federal Government officers or employees acting as part of their official duties; on a worldwide, perpetual, irrevocable, royalty-free basis to BMJ Publishing Group Ltd ("BMJ") its licensees and where the relevant Journal is co-owned by BMJ to the co-owners of the Journal, to publish the Work in this journal and any other BMJ products and to exploit all rights, as set out in our licence.

The Submitting Author accepts and understands that any supply made under these terms is made by BMJ to the Submitting Author unless you are acting as an employee on behalf of your employer or a postgraduate student of an affiliated institution which is paying any applicable article publishing charge ("APC") for Open Access articles. Where the Submitting Author wishes to make the Work available on an Open Access basis (and intends to pay the relevant APC), the terms of reuse of such Open Access shall be governed by a Creative Commons licence – details of these licences and which Creative Commons licence will apply to this Work are set out in our licence referred to above.

Other than as permitted in any relevant BMJ Author's Self Archiving Policies, I confirm this Work has not been accepted for publication elsewhere, is not being considered for publication elsewhere and does not duplicate material already published. I confirm all authors consent to publication of this Work and authorise the granting of this licence.

For peer review only

The prevalence of mental health conditions, sensory impairments, and physical disability in people with co-occurring intellectual disabilities and autism compared with other people – a cross-sectional total population study of 5,295,403 people in Scotland

Dunn, K., Rydzewska, E., Fleming, M., Cooper, S-A.

Kirsty Dunn

Department of mental health and wellbeing, Gartnavel Royal Hospital, Administration Building, 1st floor, 1055 Great Western Road, Glasgow G12 0XH, UK

Kirsty.wright@glasgow.ac.uk Tel: 0141 211 3889

Ewelina Rydzewska

Department of mental health and wellbeing, Gartnavel Royal Hospital, Administration Building, 1st floor, 1055 Great Western Road, Glasgow G12 0XH, UK

Ewelina.rydzewska@glasgow.ac.uk Tel: 0141 211 3889

Michael Fleming

Department of public health, Institute of Health and Wellbeing, University of Glasgow, 1 Lilybank Gardens, Glasgow, G12 8RZ, UK

Michael.fleming@glasgow.ac.uk Tel: +44 (0) 141 330 3415

Sally-Ann Cooper

Department of mental health and wellbeing, Gartnavel Royal Hospital, Administration Building, 1st floor, 1055 Great Western Road, Glasgow G12 0XH, UK

Sally-ann.cooper@glasgow.ac.uk Tel: +44 (0)141 211 0690/3701 Fax: +44 (0)141 211 0356

Word count: 4,009

Keywords: Intellectual disabilities, autism, mental health conditions, sensory impairments, vision, hearing, physical disability, co-morbidity.

For peer review only - http://bmjopen.bmj.com/site/about/guidelines.xhtml

Abstract

Objectives: To investigate prevalence of mental health conditions, sensory impairments, and physical disability in children, adults, and older adults with co-occurring intellectual disabilities and autism (given its frequent co-occurrence), compared with the general population.

Design: Whole country cross-sectional cohort study.

Setting: General community.

Participants: 5,709 people with co-occurring intellectual disabilities and autism (both conditions together), compared with 5,289,694 other people.

Outcome measures: Rates and odds ratios (OR) with 95% confidence intervals (95% CI) for mental health conditions, visual impairment, hearing impairment and physical disability in people with co-occurring intellectual disabilities and autism compared with other people (adjusted for age, sex, and interaction between age and co-occurring intellectual disabilities and autism).

Results: All four long-term conditions were markedly more common in children, adults, and older adults with co-occurring intellectual disabilities and autism compared with other people. For mental health, OR=130.8 (95% CI 117.1, 146.1); visual impairment OR=65.9 (95% CI 58.7, 73.9); hearing impairment OR=22.0 (95% CI 19.2, 25.2); physical disability OR=157.5 (95% CI 144.6, 171.7). These ratios are also greater than previously reported for people with *either* intellectual disabilities *or* autism (rather than co-occurring intellectual disabilities and autism)

Conclusions: We have quantified the more than double disadvantage for people with co-occurring intellectual disabilities and autism, in terms of additional mental health conditions and impairments. This may well impact on quality of life. It raises challenges for staff working with these people in view of

For peer review only - http://bmjopen.bmj.com/site/about/guidelines.xhtml

additional complexity in assessments, diagnoses, and interventions of additional health conditions, as sensory impairments and mental health conditions in particular, compound with the persons pre-existing communication and cognitive problems in this context. Planning is important, with staff being trained, equipped, resourced and prepared to address the challenge of working for people with these conditions.

Keywords: Intellectual disabilities, autism, mental health conditions, sensory impairments, vision, hearing, physical disability, co-morbidity.

Strengths and limitations of this study

- Large scale, whole country cross-sectional study, with a high response rate (94%), so the results are representative of the whole population.
- Intellectual disabilities, autism, and additional long-term conditions were enquired about systematically for everyone in the population.
- The wording of questions was tested in advance, via cognitive question testing during the design of Scotland's Census, 2011.
- Limitations include proxy-reporting.
- People known to have autism/Asperger's syndrome, intellectual disabilities, and the four long-term conditions were reported, rather than each undergoing detailed individual research assessments which are not possible in such large population studies.

For peer review only - http://bmjopen.bmj.com/site/about/guidelines.xhtml

Background

People with intellectual disabilities have more mental and physical health needs than other people.[1-4] People with autism also appear to have more mental and physical health needs than other people.[5-8] A whole population study of Scotland reported that 21.7% of people with intellectual disabilities also had autism, and 18.0% of people with autism also had intellectual disabilities,[9] so this dually diagnosed group warrant investigation. One would suspect that this population with co-occurring intellectual disabilities and autism (both conditions together) are likely to have a high level of additional health needs, but this has received little previous study. A higher number of additional health needs increases the likelihood of misdiagnosis, and treatment interactions, so requires more complex treatment plans. Hence it is important to investigate long-term additional health needs experienced by people with co-occurring intellectual disabilities and autism.

Some studies have investigated mental ill-health in people with co-occurring intellectual disabilities and autism. A small study of 149 adults with severe or profound intellectual disabilities and autism, living in state-run developmental centres in Louisiana, USA, compared comorbidity with 158 adults with intellectual disabilities without autism in the same centres. The former group had more symptomology for anxiety, mania, schizophrenia, stereotypies, self-injurious behaviour, eating disorders, sexual disorders, and impulse control.[10] A study in Norway compared 62 adults with co-occurring autism and intellectual disabilities under the care of autism services, with 132 adults with intellectual disabilities only receiving intellectual disabilities support.[11] High levels of psychiatric disorders were reported in both groups; 53.2% in the co-occurring

For peer review only - http://bmjopen.bmj.com/site/about/guidelines.xhtml

intellectual disabilities and autism group, and 17.4% in the intellectual disabilities only group. An English study of referrals to a specialist intellectual disabilities psychiatric service described 42% of the 137 referred adults who had autism as well as intellectual disabilities to have comorbid psychopathology, most commonly schizophrenia.[12] A study of youth aged 14-20 years age, gender matched 36 people with co-occurring intellectual disabilities and autism with 36 people with intellectual disabilities without autism.[13] They reported the former group to have more episodes of mental ill-health, most commonly depression. A study of Medicaid claims data 2012-2015 in Winsconsin, USA compared adults aged 40+years; 64 with autism and intellectual disabilties and 79 with autism but no intellectual disabilities.[8] They reported no statistical difference between the two groups for claims for psychiatric disorders. Claim rates were high over all (67.2% for those who additionally had intellectual disabilties, and 75.9% for those who did not).[8] The authors state the autism only sample was probably skewed towards the lower end of socioeconomic status of all people with autism.[8] A study of people aged 8-29 years with intellectual disabilities and challenging behaviour living in four residential units in England included 69 who also had autism and 13 who did not.[14] They reported a higher prevalence of organic disorders, anxiety and stereotypies in the young people with co-occurring intellectual disabilities and autism. This literature is difficult to summarise overall, as, as well as having small sample sizes, the participants were not drawn from representative populations.

A further study had the advantage of being population-based, but was still small in size.[15] It compared the prevalence, and incidence, of mental ill-health in 77 adults with co-occurring intellectual disabilities and autism with 946

For peer review only - http://bmjopen.bmj.com/site/about/guidelines.xhtml

adults with intellectual disabilities without autism, and also with 154 individually age, gender, ability-level, and Down syndrome matched controls. The adults with autism had a higher point prevalence of problem behaviours than the 946 without autism, but compared with the 154 matched controls there was no difference in prevalence, or incidence of either problem behaviours or other mental ill-health.[15] Three large whole population studies have reported that of people with intellectual disabilities, 21.7% reported mental health conditions;[3] and of people with autism, 33.0% of adults,[6] and 7.6% of children[7] reported mental health conditions, but did not report the rates for people with co-occurring intellectual disabilities and autism.

With regards to sensory impairments, of the 36 matched youth with intellectual disabilities with and without autism, 38.9% with autism reported having visual problems compared with 50.0% without autism, and 13.9% with autism reported having hearing problems compared with 19.4% without autism.[13] An intellectual disabilities register study reported that 95 of the 368 (25.8%) adults with intellectual disabilities who had visual impairment also had markers for autism, compared with 422 of 2,674 (16%) of those who had normal vision, and that 46 of the 60 (76.7%) of the adults with intellectual disabilities and congenital blindness also had markers for autism, compared with only 36 of the 67 (53.7%) with normal vision.[16]

We have not identified other papers on sensory impairments or any on physical disabilities in people with co-occurring intellectual disabilities and autism. However, previous large whole population studies have reported that of people with intellectual disabilities, 12.4% reported blindness/sight loss, 13.1% reported deafness/hearing loss, and 32.6% reported physical disability.[3]

For peer review only - http://bmjopen.bmj.com/site/about/guidelines.xhtml

Of people with autism, 12.1% of adults[6] and 3.5% of children reported blindness/sight loss,[7] 14.1% of adults[6] and 2.9% of children reported deafness/hearing loss,[,7] and 24.0% of adults[6] and 10.7% of children reported physical disability.[7] They did not, however, report the rates of these conditions for people with co-occurring intellectual disabilities and autism.

Given the frequent overlap of intellectual disabilities and autism, information on the associated comorbid conditions is important, to assist policy makers, planners, and practitioners to best adapt services for individuals with co-occurring intellectual disabilities and autism. This paper aims to investigate the prevalence of mental health conditions, sensory impairments, and physical disability in children, adults, and older adults with co-occurring intellectual disabilities and autism, compared with other people.

Methods

Approval

Approval was obtained from the Scottish Government to undertake secondary data analysis of Scotland's Census, 2011.

Data Source

Scotland's Census provides information on Scotland's population every ten years, with the most recent Census on 27th March 2011.[17] The Census provides information on the number and characteristics of Scotland's population and households on the Census date.

For peer review only - http://bmjopen.bmj.com/site/about/guidelines.xhtml

It is a legal requirement to complete the census form and households were informed that failure to make a Census return, or supplying false information could result in a £1,000 fine. A very high response rate was achieved, with an estimated 94% of all of Scotland's population completing the Census. The Census team used a Census Coverage Survey with about 40,000 households, to estimate numbers and characteristics of the missing 6%.[18] The Coverage Survey and Census records were deterministically matched to check for duplicates. Individuals estimated to have been missed were then imputed using a subset of characteristics from real individuals. The edit and imputation methodology was adapted from the Office for National Statistics rigorous and systematic guidelines.[18]

The Census was completed by the head of each household on behalf of all occupants of the household. We consider it unlikely that people with co-occurring intellectual disabilities and autism completed the form, given the reading age required to do so. Rather, we expect that the people who completed the form on their behalf were parent-carers in family households, support workers for people living in supported accommodation, and the managers/key workers at communal establishments.

Variables

The census included a question to identify people with intellectual disabilities and autism, as well as mental health conditions, sensory impairments, and physical disabilities: 'Do you have any of the following conditions which have lasted, or are expected to last, at least 12 months? Tick all that apply'. There was a choice of ten possible responses to this question: deafness or partial hearing loss; blindness or partial sight loss; learning disability (for example,

For peer review only - http://bmjopen.bmj.com/site/about/guidelines.xhtml

Down's syndrome); learning difficulty (for example, dyslexia); developmental disorder (for example, Autistic Spectrum Disorder or Asperger's Syndrome); physical disability; mental health condition; long-term illness, disease or condition; other condition. For "other condition" the option of providing more detail in an open text response was provided.

In Scotland, the term "learning disability" is used synonymously with that of "intellectual disabilities" used internationally. Importantly, the Census differentiated between intellectual disabilities and specific learning disabilities; and between intellectual disabilities and autism.

During the methodology development for Scotland's Census, 2011, cognitive question testing was undertaken on the questions on long-term health conditions and disabilities. This was to determine whether the questions were answered accurately, and to identify any changes needed to improve data quality and/or the acceptability of the way questions were phrased. Cognitive interviewing is a widely used approach to critically evaluate and improve survey questionnaires.[19] This approach enables researchers to modify survey material to enhance clarity. Retrospective probing was conducted with 102 participants with a variety of sex, age, and health conditions and disabilities (including people with more than one of the conditions). They included people with autism, intellectual disabilities, dyslexia, dyspraxia, speech impairment, mental health conditions (both milder and more serious), and other long-term conditions.[20] Using the cognitive interviewing results, the question aimed to detect autism was improved and rephrased, to better capture this information. The questions on intellectual disability, mental health condition, visual

For peer review only

impairment, hearing impairment, and physical disability did not require any modifications.

The Census team imputed answers for the 14.7% who did not tick any of the boxes in question on long-term conditions, based on their free text answers for this question and answers to other health questions in the Census, which increased the completion rate to 97.4%. For the remaining 2.6%, the Census team assumed the most plausible explanation was that the person had no long-term condition but did not see the “No condition” check box at the end of the question, and hence recorded them as having no conditions.

For peer review only

Procedure

Data was downloaded from the National Records of Scotland Census data archive.

Data Analysis

We calculated the number and rate per 1,000 population of children and adults with co-occurring intellectual disabilities and autism. We then calculated the number and percentage of people with mental health conditions, deafness or partial hearing loss, blindness or partial sight loss, and physical disability, for those with co-occurring intellectual disabilities and autism, compared with individuals who do not have co-occurring intellectual disabilities and autism using chi-squared (χ^2) tests. For the whole population we then used logistic regression to calculate the odds ratios (OR: 95% confidence interval, 95% CI) to estimate the effect size of the primary interest, co-occurring intellectual disabilities and autism, on the binary response of having each of the four specific types of long-term health conditions, adjusted for age and sex. Sex was

For peer review only - http://bmjopen.bmj.com/site/about/guidelines.xhtml

binary, with male as the reference group. Age was categorised into groups: 0-15, 16-24, 25-34, 35-44, 45-54, 55-64, 65-74, 75+, with 0-15 years as the reference group. We repeated the regressions, including the interaction term of age x co-occurring intellectual disabilities and autism, as people with the most severe disabilities die earlier, which may affect the profile of additional health problems differently to that seen in the general population. The same reference groups were used. All analysis was conducted using SPSS software version 22.

Patient and public involvement

The Scottish Learning Disabilities Observatory, where this research was undertaken, has a specific remit for people with intellectual disabilities and people with autism. Its steering group includes partners from third sector organisations and experts by experience, who approved the workplan for this project prior to it commencing. Results from this study will be disseminated for people with intellectual disabilities and autism in an easy-read version via the Scottish Learning Disabilities Observatory website, newsletters, and conference.

Results

Characteristics of the Sample

Scotland's Census, 2011, includes records on 5,295,403 people aged 0-75+ years. 5,709/5,295,403 (1.08/1,000) people had co-occurring intellectual disabilities and autism; of whom 3,769 (66.0%) were male and 1,940 (44.0%) were female. Overall, 2,362/916,331 (2.58/1,000) of the total population of

For peer review only

children (0-15 years), and 3,347/4,379,072 (0.76/1,000) adults (16-75+ years) had co-occurring intellectual disabilities and autism.

Compared with the population who did not have co-occurring intellectual disabilities and autism, the population with co-occurring intellectual disabilities and autism had more males (66.0% versus 48.5%; χ^2=703.5; df=1; p<0.001); were younger (χ^2=3894.7; df=7; p<0.001); were more likely to have been born in the UK rather than elsewhere (χ^2=101.9; df=1; p<0.001), revealing lesser geographic mobility; and were no different with regards to Caucasian versus non-Caucasian ethnicity (χ^2=1.1; df=1; p=0.3) (table 1).

- Insert table 1 about here -

Long-term Health Conditions

Table 2 shows the proportion of people with co-occurring intellectual disabilities and autism, who had each of the four additional long-term health conditions, compared to people who did not have co-occurring intellectual disabilities and autism. Some people in the sample had more than one long-term health condition.

- Insert table 2 about here -

Mental Health Condition

Adjusting for age and sex, given the different distributions compared with the general population, having co-occurring intellectual disabilities and autism had an OR=25.553 (23.933-27.282, 95% CI) for mental health conditions (table 3). When the interaction term was added (age x co-occurring intellectual

For peer review only - http://bmjopen.bmj.com/site/about/guidelines.xhtml

disabilities and autism), co-occurring intellectual disabilities and autism had an OR=130.803 (117.131-146.07048.8, 95% CI) for a mental health condition (table 3).

- Insert table 3 about here -

Blindness or partial sight loss

Adjusting for age and sex, having co-occurring intellectual disabilities and autism had an OR=36.781 (34.212-39.542, 95% CI) for blindness or partial sight loss (table 4). When the interaction term was added (age x co-occurring intellectual disabilities and autism), co-occurring intellectual disabilities and autism had an OR=65.897 (58.743-73.922, 95% CI) for blindness or partial sight loss (table 4).

- Insert table 4 about here -

Deafness or partial hearing loss

Adjusting for age and sex, having co-occurring intellectual disabilities and autism had an OR=11.331 (10.430-12.309, 95% CI) for deafness or partial hearing loss (table 5). When the interaction term was added (age x co-occurring intellectual disabilities and autism), co-occurring intellectual disabilities and autism had an OR=21.996 (19.196-25.205, 95% CI) for deafness or partial hearing loss (table 5).

- Insert table 5 about here -

Physical disability

For peer review only - http://bmjopen.bmj.com/site/about/guidelines.xhtml

Adjusting for age and sex, having co-occurring intellectual disabilities and autism had an OR=61.159 (57.601-64.938, 95% CI) for physical disability (table 6). When the interaction term was added (age x co-occurring intellectual disabilities and autism), co-occurring intellectual disabilities and autism had an OR=157.535 (144.577-171.655, 95% CI) for physical disability (table 6).

- Insert table 6 about here –

Prevalence differences

Prevalence differences are apparent from table 2. For children and young people aged 0-15 years, the difference in prevalence between the population with co-occurring intellectual disabilties and autism, and the rest of the general population, was: 20.0/100 for mental health conditions, 16.2/100 for blindness/partial sight loss, 9.8/100 for deafness/partial hearing loss, and 44.9/100 for physical disability. For adults aged 16-64 years, the difference in prevalence between the population with co-occurring intellectual disabilties and autism, and the rest of the general population, was: 31.5/100 for mental health conditions, 17.3/100 for blindness/partial sight loss, 12.4/100 for deafness/partial hearing loss, and 34.8/100 for physical disability. For older adults aged 65+ years, the difference in prevalence between the population with co-occurring intellectual disabilties and autism, and the rest of the general population, was: 61.0/100 for mental health conditions, 52.9/100 for blindness/partial sight loss, 37.7/100 for deafness/partial hearing loss, and 55.3/100 for physical disability.

Discussion

For peer review only - http://bmjopen.bmj.com/site/about/guidelines.xhtml

Principle findings

Mental health conditions, blindness or partial sight loss, deafness or partial hearing loss, and physical disability were all significantly more common in people with co-occurring intellectual disabilities and autism than people without these co-occurring conditions. The odds ratios after adjusting for age and sex and the interaction term, were substantial, being 131, 66, 22, and 158 respectively. This is important as each of these conditions are disabling and can significantly impact an individual's quality of life. They contribute to high rates of multi-morbidity, which, on top of communication and cognitive problems due to autism and intellectual disabilities, renders assessments, diagnosis, and treatment of additional health problems more complex than for other people.

Across all age groups, blindness, deafness, and physical disability were more common in females then males with co-occurring intellectual disabilities and autism, unlike the sex ratios in people without co-occurring intellectual disabilities and autism. Mental health conditions were more common in males than females with co-occurring intellectual disabilities and autism, except for the 65+ year group, contrary to the gender ratios in other people. All conditions were more prevalent with increasing age in the people with co-occurring intellectual disabilities and autism, except for physical disability which was more common in the children/youth and older people than in the adults.

There are likely to be many biological, social, and environmental reasons accounting for these results.

Comparison with existing literature

For peer review only - http://bmjopen.bmj.com/site/about/guidelines.xhtml

The prevalence of these additional long-term health conditions has seldom been investigated in people with co-occurring intellectual disabilities and autism, particularly in comparison with other people, and never, to our knowledge, as a total population study. While prevalence rates of mental health conditions and impairments in a full country population for individuals with intellectual disabilities, and separately for individuals with autism have been compared to the general population, no such study has been conducted on the prevalence of mental health conditions and impairments for those with co-occurring intellectual disabilities and autism. We found all of the long-term health conditions were more common than in those without co-occurring intellectual disabilities and autism.

Smaller, less representative studies have reported a higher rate of mental health conditions in adults and youth with co-occurring intellectual disabilities and autism compared with those with intellectual disabilities and without autism,[9-14] but not all.[15] People with autism have been reported to have more mental health conditions than other people (OR=9 in adults and OR=16 in children),[6,7] as have people with intellectual disabilities compared with other people (OR=7),[3] using the same Scotland's Census, 2011 data as in this current paper, whereas the comparable ratio we now report for people with co-occurring intellectual disabilities and autism for mental health conditions was OR=26. Having the co-occurring conditions therefore presents a much higher risk of mental health conditions than either intellectual disabilities or autism on their own.

The previous small study of youth reported lower rates of visual and hearing impairments in those with co-occurring intellectual disabilities and autism

For peer review only - http://bmjopen.bmj.com/site/about/guidelines.xhtml

(38.9% and 13.9%) compared with those with intellectual disabilities but without autism (50% and 19.4%).[13] This was in contrast with the larger study reporting more autistic symptoms in adults with intellectual disabilities and visual impairments than in adults with intellectual disabilities but without visual impairments.[16] Adults with autism have been reported to have more blindness or partial sight loss, and deafness or partial hearing loss than other people (12.1% and 17.5%),[6] as have children with autism (3.5% and 2.9%),[7] and people (children and adults combined) with intellectual disabilities compared with other people (13.1% and 12.4%),[3] using the same Scotland's Census 2011 as in this current paper. This current study found the comparable rates for people with co-occurring intellectual disabilities and autism for blindness or partial sight loss, and deafness or partial hearing loss was 21.7% and 19.3% for adults, and 16.6% and 10.3% for children. Having the co-occurring conditions of intellectual disabilities and autism therefore presents a much higher risk of sensory impairments than for children and adults with autism, and for people with intellectual disabilities (although children were not separately studied in the previous report).

Regarding physical disability, 32.6% of people with intellectual disabilities were previously reported to have physical disability.[3] Of people with autism, 24.0% of adults and 10.7% of children reported physical disability.[6,7] These rates are lower than those we report in this current study of people with co-occurring intellectual disabilities and autism – 45.6% of children and 42.2% of adults. Cerebral palsy is associated with intellectual disabilities, but the extent of this association does not appear to account for the differences found in physical disability: cerebral palsy has been reported to occur in 13% of children and

For peer review only - http://bmjopen.bmj.com/site/about/guidelines.xhtml

young people with intellectual disabilities,[21] and in 3.2% of general population children and young people in the USA.[22]

Strengths and limitations

Strengths of the study include its large scale and general population comparison group, whole population coverage, and very high response rate so the results are representative of the whole population. Intellectual disabilities, autism, and the long-term health conditions were enquired about systematically for everyone in the population. We believe the results are therefore generalisable to other high-income countries. The cognitive question testing during the design of the Census is a further strength. The Census had clear categories to distinguish between intellectual disabilities, specific learning disability (like dyslexia), and autism.

Limitations include the proxy-reporting, which may, or may not reflect self-reports. Without proxy-reports, we would have no information on people unable to self-report due to their disabilities, and a previous review on the topic concluded that overall, proxy reports are a useful addition to determine aspects of well-being in people with intellectual disabilities.[23] Additionally, people were reported who were known to have autism/Asperger's syndrome, intellectual disabilities, and the additional long-term health conditions, rather than detailed individual research assessments being undertaken which are clearly not possible in such large population studies, and may therefore be subject to a degree of error which we were not able to check.

Scotland's Census 2011 was administered 8 years ago, and so any potential changes in prevalence of conditions since then are not captured by our

For peer review only - http://bmjopen.bmj.com/site/about/guidelines.xhtml

analyses. People with intellectual disabilities and autism are both higher health care users than other people and so may receive more diagnoses, but they are also subject to “diagnostic overshadowing”. We do not know the extent to which these factors may impact on reporting of mental health conditions, sensory impairments, and physical disability at Scotland’s Census, 2011. However, given the long-term nature of these conditions, any impact is likely to be less than it would be for acute conditions.”

The study is cross-sectional, rather than longitudinal. We acknowledge that the use of odds ratios may overestimate the strength of associations in cross-sectional studies where the prevalence in the general population is very low; calculating odds ratios has enabled us to draw comparisons with previously published results on mental health conditions and impairments of people with intellectual disabilities, and of people with autism. We also present example prevalence differences. It is also important to note that whilst our regressions adjusted for sex and age, the effect sizes for sex and age shown in tables 3-6 might not be the total effect of sex and age on the four outcomes: they show the proportion of the sex, age effect on the odds ratio for the four outcomes that are not mediated through any sex, age effect on co-occurring intellectual disabilities and autism.[24] Hence in our discussions of sex and age, we referred to prevalence rather than odds ratios.

Implications

Management of individuals with multiple health conditions or disabilities presents significant challenges for health care practitioners. A review of 123 studies on care management for individuals with multiple chronic conditions in the USA reported that these patients access services more frequently and use

For peer review only - http://bmjopen.bmj.com/site/about/guidelines.xhtml

a larger range of services than other patients, making the coordination of their care more difficult and often leading to suboptimal care.[25] Evidence from the UK suggests that individuals with intellectual disabilities and additional long term conditions receive poorer management than members of the general population with the same conditions.[26]

We have shown there is a greater than double disadvantage for people with co-occurring intellectual disabilities and autism, in terms of additional long-term health conditions. We found that, and quantified, mental health conditions, sensory impairments, and physical disabilities are more common than people without co-occurring intellectual disabilities and autism, and in people with just intellectual disabilities or just autism. This may well impact on quality of life. It raises challenges for staff working with people with these co-occurring conditions in view of the additional complexity in assessments, diagnoses, and interventions, as sensory impairments and mental health conditions in particular, interact with the persons pre-existing communication and cognitive problems in this context. Therefore, it is important that these co-occurring conditions are planned for with staff being trained, equipped, resourced and prepared to address the challenge. At present, Government policy and strategy in Scotland addresses needs of people with intellectual disabilities,[27] and autistic people,[28] but has not yet established a framework for people with these co-occurring conditions; our findings are therefore highly relevant.

References

For peer review only - http://bmjopen.bmj.com/site/about/guidelines.xhtml

1. Cooper S-A, McLean G, Guthrie B, McConnachie A, Mercer S, Sullivan F, Morrison. J. Multiple physical and mental health comorbidity in adults with intellectual disabilities: population-based cross-sectional analysis. *BMC Family Practice* 2015; 16: 110.
2. Hughes-McCormack LA, Rydzewska E, Henderson A., Rintoul J, Macintyre C, Cooper S-A. Prevalence and general health status of people with intellectual disabilities in Scotland -a total population study. *J Epidemiol Community Health 2018;* 72: 78–85.
3. Hughes-McCormack LA, Rydzewska E, Henderson A, Rintoul J, Macintyre C, Cooper S-A. Prevalence of mental ill-health and relationship with physical health in a whole country population of 26,349 people with intellectual disabilities, compared with the general population. *Br J Psychiatry Open,* 2017*;* 3: 243-248.
4. Kinnear D, Morrison J, Allan L, Henderson A, Smiley E, & Cooper S-A. Prevalence of physical conditions and multimorbidity in a cohort of adults with intellectual disabilities with and without Down syndrome: cross-sectional study. *BMJ Open* 2018; *8*:e018292.
5. Croen LA, Zerbo O, Qian Y, Massolo ML, Rich S, Sidney S, Kripke C. The health status of adults on the autism spectrum. *Autism* 2015; 19:814–823.
6. Rydzewska E, Hughes-McCormack LA, Gillberg C, Henderson A, MacIntyre C, Rintoul J, & Cooper S-A. Prevalence of long-term health conditions in adults with autism: observational study of a whole country population. *BMJ Open* 2018a; 8(8): e023945.
7. Rydzewska E, Hughes-McCormack LA, Gillberg C, Henderson A, MacIntyre C, Rintoul J, Cooper S-A. Prevalence of sensory impairments, physical and intellectual disabilities, and mental health in children and young people

For peer review only - http://bmjopen.bmj.com/site/about/guidelines.xhtml

with self/proxy-reported autism - observational study of a whole country population. *Autism* 2018; 23:1201-9.

8. Bishop-Fitzpatrick L, Rubenstein E. The physical and mental health of middle aged and older adults on the autism spectrum and the impact of intellectual disability. *Res Autism Spectrum Dis* 2019; 63:34-41.
9. Dunn K, Rydzewska E, Macintyre C, Rintoul J, & Cooper S-A. The prevalence and general health status of people with intellectual disabilities and autism co-occurring together: a total population study. *J Inellectual Disabilit Res* 2018, 63: 277-285.
10. Cervantes PE, Matson JL. Comorbid symptomology in adults with autism spectrum disorder and intellectual disability. *J Autism Dev Disord* 2015; *45(12)*: 3961-3970.
11. Bakken TL, Helverschou SB, Eilertsen DE, Heggelund T, Myrbakk E, & Martinsen, H. Psychiatric disorders in adolescents and adults with autism and intellectual disability: A representative study in one county in Norway. *Res Dev Disabil* 2010; *31*: 1669-1677.
12. Tsakanikos E, Sturmey P, Costello H, Holt G, Bouras N. Referral trends in mental health services for adults with intellectual disability and autism spectrum disorders. *Autism* 2007;11:9-17.
13. Bradley E, Bolton P. Episodic psychiatric disorders in teenagers with learning disabilities with and without autism. *Br J Psychiatr* 2006; 189:361-6.
14. Hill J, Furniss F. Patterns of emotional and behavioural disturbance associated with autistic traits in young people with severe intellectual disabilities and challenging behaviours. *Res Dev Disabil* 2006; 27:517-28.
15. Melville CA, Cooper S-A, Morrison J, Smiley E, Allan L, Jackson A, Finlayson J, Mantry D. The prevalence and incidence of mental ill-health in adults

For peer review only

For peer review only - http://bmjopen.bmj.com/site/about/guidelines.xhtml

with autism and intellectual disabilities. *J Autism Dev Disord* 2008; 38:1676-88.

16. Kiani, R, Bhaumik, S, Tyrer, F, Bankart, J, Miller, H, Cooper, S-A, Brugha, T. The relationship between autism spectrum disorder and visual impairments among adults with intellectual disability. *Autism Res 2019;* 12:1411-1422.

17. National Records of Scotland. Scotland's Census, 2011. Supporting information. *National Records of Scotland*. 2011 http://www.scotlandscensus.gov.uk/supporting-information.

18. Office for National Statistics. Survey Methodology Bulletin No. 69 – September 2011. 2011 https:// webarchive.nationalarchives.gov.uk/20160108193745/http://www. ons.gov.uk/guide-method/method-quality/survey-methodologybulletin/smb-69/index.html.

19. Willis GB. Cognitive interviewing: A tool for improving questionnaire design. Sage Publications; 2004.

20. General Register Office for Scotland. 2011 Census Question Testing- The Health and Disability Questions (2009). 2009 https://www.scotlandscensus.gov.uk/documents/research/2011-census-healthdisability-questions.pdf.

21. Van Naarden Braun K, Christensen D, Doemberg N, Schieve L, Rice C, Wiggins L, Schendel , Yeargin-Allsopp M Trends in the prevalence of autism spectrum disorder, cerebral palsy, haring loss, intellectual disability, and vision impairment, metropolitan Atlanta, 1999-2010. *PLOSOne* https://doi.org/10.1371/jornal.pone.0124120

22. McGuire DO, Tian LH, Yeargin-Allsop M, Dowling NF, Christensen DL. Prevalence of cerebral palsy, intellectual disability, hearing in loss, and

For peer review only - http://bmjopen.bmj.com/site/about/guidelines.xhtml

blindness, National Health Interview Survey, 2009-2016. *Disabil Health J* 2019; 12:443-451.

23. Perkins EA, Berkman KA. Into the unknown: Aging with autism spectrum disorders. *Am J Intellect Dev Disabil* 2012; 117:478-96.

24. Westreich D, Greenland, S. The table 2 fallacy: presenting and interpreting confounder and modifier coefficients. *Am J Epidemiology* 2013; 177:292-298.

25. Vogeli C, Shields AE, Lee TA, Gibson TB, Marder WD, Weiss KB, Blumenthal D. Multiple chronic conditions: prevalence, health consequences, and implications for quality, care management, and costs. *J Gen Internal Med* 2007; 22(3):391-5.

26. Cooper S-A, Hughes-McCormack L, Greenlaw N, McConnachie A, Allan L, Baltzer M, McArthur L, Henderson A, Melville C, McSkimming P, Morrison J. Management and prevalence of long-term conditions in primary health care for adults with intellectual disabilities compared with the general population: A population-based cohort study. *J Applied Res Intellectual Disabil* 2018; 31:68-81.

27. Scottish Government. The keys to life: Unlocking futures for People with Learning Disabilities Implementation framework and priorities 2019-2021. 2019; https://keystolife.info/wp-content/uploads/2019/03/Keys-To-Life-Implementation-Framework.pdf

28. Scottish Government. Scottish Strategy for Autism: outcomes and priorities 2018-2021. 2018; https://www.gov.scot/publications/scottish-strategy-autism-outcomes-priorities-2018-2021/

Funding

For peer review only - http://bmjopen.bmj.com/site/about/guidelines.xhtml

This work was supported by the UK Medical Research Council, grant number: MC_PC_1717), and the Scottish Government via the Scottish Learning Disabilities Observatory.

The study sponsor and funders had no role in the study design; in the collection, analysis, and interpretation of data; in the writing of the report; and in the decision to submit the article for publication.

The researchers are independent from the funders.

Competing interests

The authors declare no competing interests.

Author's contributions

KD analysed and interpreted the data, and wrote the first draft of the manuscript. ER contributed to data access, data interpretation, and drafting the manuscript. MF provided advice on response to reviewer comments regarding choice of statistical methods and contributed to the writing of the manuscript. S-AC conceived and managed the project, interpreted data, and contributed to writing the manuscript. All approved the final version of the manuscript. S-AC is the study guarantor.

Data sharing

For peer review only - http://bmjopen.bmj.com/site/about/guidelines.xhtml

Data is available via National Records of Scotland, following project approval.

Data are available at the following link

https://www.scotlandscensus.gov.uk/ods-web/data-warehouse.html#additionaltab

Acknowledgments

We thank the National Records of Scotland Census team, and the Scottish population who completed the Census.

For peer review only

For peer review only - http://bmjopen.bmj.com/site/about/guidelines.xhtml

Table 1: Characteristics of people with and without, co-occurring intellectual disabilities and autism

	**People without co-occurring intellectual disabilities and autism**<br>N=5,289,694 (100%)<br>Number (%)	**People with co-occurring intellectual disabilities and autism**<br>N=5,709 (100%)<br>Number (%)
**Gender***		
Males	2,563,675 (48.5%)	3,769 (66.0%)
Females	2,726,019 (51.5%)	1,940 (44.0%)
**Age groups***		
0-15	913,969 (17.3%)	2,362 (41.4%)
16-24	631,094 (11.9%)	1,394 (24.4%)
25-34	666,725 (12.6%)	602 (10.5%)
35-44	734,304 (13.9%)	450 (7.9%)
45-54	786,355 (14.9%)	401 (7.0%)
55-64	667,157 (12.6%)	256 (4.5%)
65+	890,090 (16.8%)	244 (4.3%)
**Country of birth***		
UK	4,920,614 (93.0%)	5,505 (96.4%)
Other Europe	172,160 (3.3%)	83 (1.5%)
Africa	46,708 (0.9%)	34 (0.6%)
Middle East and Asia	104,480 (2.0%)	50 (0.9%)
The Americas and the Caribbean	33,325 (0.6%)	28 (0.5%)
Other	12,407 (0.2%)	9 (0.2%)
**Ethnicity**		
White	5,078,910 (96.0%)	5,497 (96.3%)
Asian	140,542 (2.7%)	136 (2.4%)
Mixed/multiple ethnicities	19,775 (0.4%)	40 (0.7%)

For peer review only - http://bmjopen.bmj.com/site/about/guidelines.xhtml

African	29,615 (0.6%)	23 (0.4%)
Caribbean or black	6,536 (0.1%)	4 (0.1%)
Other ethnic groups	14,316 (0.3%)	9 (0.2%)

*People with co-occurring intellectual disabilities and autism versus people without co-occurring intellectual disabilities and autism; p<0.01

For peer review only

For peer review only - http://bmjopen.bmj.com/site/about/guidelines.xhtml

Table 2: Prevalence of comorbidities in people with and without co-occurring intellectual disabilities and autism by age and sex

People with co-occurring intellectual disabilities and autism									
**Condition**	**Children/youth, 0-15 years** N=2,362			**Adults, 16-64 years** N=3,103			**Older people, 65+ years** N=244		
	**M** N=1,563 (100%)	**F** N=799 (100%)	**Total** N=2,362 (100%)	**M** N= 2,073 (100%)	**F** N= 1,030 (100%)	**Total** N= 3,103 (100%)	**M** N= 133 (100%)	**F** N= 111 (100%)	**Total** N= 244 (100%)
**Mental health condition**	328 (21.0%)	152 (19.0%)	480 (20.3%)	768 (37.0%)	377 (36.6%)	1,145 (36.9%)	80 (60.2%)	80 (72.1%)	160 (65.6%)
**Blindness/partial sight loss**	214 (13.7%)	177 (22.2%)	391 (16.6%)	355 (17.1%)	220 (21.4%)	575 (18.5%)	71 (53.3%)	80 (72.1%)	151 (61.9%)
**Deafness/partial hearing loss**	148 (9.5%)	95 (11.9%)	243 (10.3%)	301 (14.5%)	190 (18.4%)	491 (15.8%)	73 (54.9%)	81 (73.0%)	154 (63.1%)
**Physical disability**	618 (39.5%)	458 (57.3%)	1,076 (45.6%)	719 (34.7%)	508 (49.3%)	1,227 (39.5%)	86 (64.7%)	99 (89.2%)	185 (75.8%)
**People without co-occurring intellectual disabilities and autism**									
**Condition**	**Children/youth, 0-15 years** N=913,969			**Adults, 16-64 years** N=3,485,635			**Older people, 65+ years** N=890,090		
	**M** N=467,543 (100%)	**F** N=446,426 (100%)	**Total** N= 913,969 (100%)	**M** N= 1,712,526 (100%)	**F** N= 1,773,109 (100%)	**Total** N= 3,485,635 (100%)	**M** N= 383,606 (100%)	**F** N= 506,484 (100%)	**Total** N= 890,090 (100%)
**Mental health condition**	1,861 (0.4%)	980 (0.2%)	2,841 (0.3%)	92,308 (5.4%)	95,108 (5.4%)	187,416 (5.4%)	14,760 (3.8%)	26,141 (5.2%)	40,901 (4.6%)
**Blindness/partial sight loss**	1,793 (0.4%)	1,439 (0.3%)	3,232 (0.4%)	24,129 (1.4%)	16,954 (1.0%)	41,083 (1.2%)	30,389 (7.9%)	49,839 (9.8%)	80,228 (9.0%)
**Deafness/partial hearing loss**	2,731 (0.6%)	2,225 (0.5%)	4,956 (0.5%)	70,543 (4.1%)	48,727 (2.7%)	119,270 (3.4%)	111,447 (29.1%)	114,393 (22.6%)	225,840 (25.4%)
**Physical disability**	3,637 (0.8%)	2,799 (0.6%)	6,436 (0.7%)	81,655 (4.8%)	82,968 (4.7%)	164,623 (4.7%)	73,759 (19.2%)	109,103 (21.5%)	182,862 (20.5%)

For peer review only - http://bmjopen.bmj.com/site/about/guidelines.xhtml

Table 3: Effect of co-occurring intellectual disabilities and autism on mental health conditions in the whole population, adjusted for sex and age

**Characteristic**		**Regression 1**		**Regression 2 (including the interaction term: age x co-occurring intellectual disabilities and autism)**	
		**Odds ratio**	**95% confidence interval**	**Odds ratio**	**95% confidence interval**
Co-occurring intellectual disabilities and autism	Not present (reference)	-	-	-	-
	Co-occurring intellectual disabilities and autism	25.553	23.933-27.282	130.803	117.131-146.070
Sex	Male (reference)	-	-	-	-
	Female	1.275	1.264-1.286	1.275	1.264-1.286
Age	0-15 (reference)	-	-	-	-
	16-24	9.449	9.039-9.877	11.322	10.781-11.890
	25-34	21.168	20.285-22.090	25.305	24.134-26.532
	35-44	29.725	28.497-31.004	35.493	33.866-37.199
	45-54	29.186	27.983-30.441	34.835	33.239-36.507
	55-64	22.633	21.633-23.617	26.997	25.750-28.304
	65+	19.319	18.518-20.155	23.005	21.947-24.115
Age x both intellectual disabilities and autism	0-15 (reference)	-	-	-	-
	16-24	-	-	0.152	0.129-0.178
	25-34	-	-	0.101	0.083-0.123
	35-44	-	-	0.083	0.067-0.104
	45-54	-	-	0.089	0.071-0.112
	55-64	-	-	0.118	0.090-0.154
	65+	-	-	0.308	0.238-0.423
Constant	-	0.002	-	0.002	-

For peer review only

Table 4: Effect of co-occurring intellectual disabilities and autism on blindness/partial sight loss in the whole population, adjusted for sex and age

Characteristic		Regression 1		Regression 2 (including the interaction term: age x co-occurring intellectual disabilities and autism)	
		Odds ratio	95% confidence interval	Odds ratio	95% confidence interval
Co-occurring intellectual disabilities and autism	Not present (reference)	-	-	-	-
	Co-occurring intellectual disabilities and autism	36.781	34.212-39.542	65.897	58.743-73.922
Sex	Male (reference)	-	-	-	-
	Female	1.011	0.999-1.022	1.011	0.999-1.022
Age	0-15 (reference)	-	-	-	-
	16-24	1.557	1.482-1.636	1.663	1.579-1.751
	25-34	1.824	1.739-1.913	1.914	1.821-2.011
	35-44	2.548	2.439-2.663	2.687	2.567-2.813
	45-54	4.416	4.241-4.599	4.668	4.475-4.870
	55-64	7.503	7.215-7.803	7.928	7.609-8.260
	65+	31.064	29.947-32.222	32.750	31.512-34.036
Age x both intellectual disabilities and autism	0-15 (reference)	-	-	-	-
	16-24	-	-	0.433	0.354-0.528
	25-34	-	-	0.669	0.521-0.844
	35-44	-	-	0.529	0.411-0.681
	45-54	-	-	0.368	0.286-0.474
	55-64	-	-	0.278	0.208-0.372
	65+	-	-	0.250	0.188-0.331
Constant	-	0.003	-	0.003	-

For peer review only - http://bmjopen.bmj.com/site/about/guidelines.xhtml

Table 5: Effect of co-occurring intellectual disabilities and autism on deafness/partial hearing loss in the whole population, adjusted for sex and age

**Characteristic**		**Regression 1**		**Regression 2 (including the interaction term: age x co-occurring intellectual disabilities and autism)**	
		**Odds ratio**	**95% confidence interval**	**Odds ratio**	**95% confidence interval**
Co-occurring intellectual disabilities and autism	Not present (reference)	-	-	-	-
	Co-occurring intellectual disabilities and autism	11.331	10.430-12.309	21.996	19.196-25.205
Sex	Male (reference)	-	-	-	-
	Female	0.687	0.682-0.693	0.688	0.682-0.693
Age	0-15 (reference)	-	-	-	-
	16-24	1.557	1.496-1.621	1.588	1.524-1.654
	25-34	2.358	2.274-2.446	2.408	2.321-2.499
	35-44	4.237	4.099-4.379	4.347	4.203-4.495
	45-54	8.546	8.285-8.815	8.769	8.496-9.051
	55-64	18.761	18.204-19.336	19.243	18.659-19.845
	65+	69.646	67.633-71.719	71.378	69.269-73.552
Age x both intellectual disabilities and autism	0-15 (reference)	-	-	-	-
	16-24	-	-	0.633	0.507-0.790
	25-34	-	-	0.735	0.569-0.948
	35-44	-	-	0.394	0.296-0.523
	45-54	-	-	0.288	0.219-0.378
	55-64	-	-	0.215	0.159-0.289
	65+	-	-	0.222	0.165-0.298
Constant	-	0.006	-	0.006	-

For peer review only - http://bmjopen.bmj.com/site/about/guidelines.xhtml

Table 6: Effect of co-occurring intellectual disabilities and autism on physical disability in the whole population, adjusted for sex and age

Characteristic		Regression 1		Regression 2 (including the interaction term: age x co-occurring intellectual disabilities and autism)	
		**Odds ratio**	**95% confidence interval**	**Odds ratio**	**95% confidence interval**
Co-occurring intellectual disabilities and autism	Not present (reference)	-	-	-	-
	Co-occurring intellectual disabilities and autism	61.159	57.601-64.938	157.535	144.577-171.655
Sex	Male (reference)	-	-	-	-
	Female	1.063	1.055-1.070	1.063	1.055-1.070
Age	0-15 (reference)	-	-	-	-
	16-24	1.440	1.388-1.495	1.573	1.513-1.637
	25-34	2.603	2.519-2.690	2.857	2.760-2.958
	35-44	5.869	5.699-6.043	6.465	6.267-6.670
	45-54	10.606	10.312-10.908	11.662	11.318-12.017
	55-64	20.730	20.166-21.310	22.756	22.096-23.436
	65+	43.680	42.517-44.875	47.894	46.530-49.298
Age x both intellectual disabilities and autism	0-15 (reference)	-	-	-	-
	16-24	-	-	0.410	0.355-0.472
	25-34	-	-	0.344	0.287-0.414
	35-44	-	-	0.123	0.100-0.152
	45-54	-	-	0.075	0.061-0.094
	55-64	-	-	0.044	0.034-0.057
	65+	-	-	0.050	0.039-0.066
Constant	-	0.006	-	0.005	-

For peer review only - http://bmjopen.bmj.com/site/about/guidelines.xhtml

STROBE Statement—Checklist of items that should be included in reports of ***cross-sectional studies***

	Item No	Recommendation
**Title and abstract**	1	(*a*) Indicate the study's design with a commonly used term in the title or the abstract<br>Page 1 (title)
		(*b*) Provide in the abstract an informative and balanced summary of what was done and what was found<br>Page 2
**Introduction**		
Background/rationale	2	Explain the scientific background and rationale for the investigation being reported<br>Pages 4-7
Objectives	3	State specific objectives, including any pre-specified hypotheses<br>Page 7, line 9
**Methods**		
Study design	4	Present key elements of study design early in the paper<br>Page 8-11
Setting	5	Describe the setting, locations, and relevant dates, including periods of recruitment, exposure, follow-up, and data collection<br>Page 7-8
Participants	6	(*a*) Give the eligibility criteria, and the sources and methods of selection of participants<br>Page 7-8
Variables	7	Clearly define all outcomes, exposures, predictors, potential confounders, and effect modifiers. Give diagnostic criteria, if applicable<br>Page 8-10
Data sources/ measurement	8*	For each variable of interest, give sources of data and details of methods of assessment (measurement). Describe comparability of assessment methods if there is more than one group<br>Page 7-10
Bias	9	Describe any efforts to address potential sources of bias<br>Page 9-10
Study size	10	Explain how the study size was arrived at<br>Page 7-8
Quantitative variables	11	Explain how quantitative variables were handled in the analyses. If applicable, describe which groupings were chosen and why<br>Page 10-11
Statistical methods	12	(*a*) Describe all statistical methods, including those used to control for confounding<br>Page 10-11
		(*b*) Describe any methods used to examine subgroups and interactions<br>Page 10-11
		(*c*) Explain how missing data were addressed<br>Page 10
		(*d*) If applicable, describe analytical methods taking account of sampling strategy<br>N/A
		(*e*) Describe any sensitivity analyses<br>N/A

Results

For peer review only - http://bmjopen.bmj.com/site/about/guidelines.xhtml

Participants	13*	(a) Report numbers of individuals at each stage of study—eg numbers potentially eligible, examined for eligibility, confirmed eligible, included in the study, completing follow-up, and analysed<br>Page 11-12
		(b) Give reasons for non-participation at each stage<br>N/A
		(c) Consider use of a flow diagram<br>N/A
Descriptive data	14*	(a) Give characteristics of study participants (eg demographic, clinical, social) and information on exposures and potential confounders<br>Page 11-12, Table 1
		(b) Indicate number of participants with missing data for each variable of interest<br>Page 10
Outcome data	15*	Report numbers of outcome events or summary measures<br>Page 13-15
Main results	16	(*a*) Give unadjusted estimates and, if applicable, confounder-adjusted estimates and their precision (eg, 95% confidence interval). Make clear which confounders were adjusted for and why they were included<br>Page 12-14, Tables 3-6
		(*b*) Report category boundaries when continuous variables were categorized<br>N/A
		(*c*) If relevant, consider translating estimates of relative risk into absolute risk for a meaningful time period<br>N/A
Other analyses	17	Report other analyses done—eg analyses of subgroups and interactions, and sensitivity analyses<br>Page 12-14
**Discussion**		
Key results	18	Summarise key results with reference to study objectives<br>Page 15-17
Limitations	19	Discuss limitations of the study, taking into account sources of potential bias or imprecision. Discuss both direction and magnitude of any potential bias<br>Page 18-20
Interpretation	20	Give a cautious overall interpretation of results considering objectives, limitations, multiplicity of analyses, results from similar studies, and other relevant evidence<br>Page 15-20
Generalisability	21	Discuss the generalisability (external validity) of the study results<br>Page 18
**Other information**		
Funding	22	Give the source of funding and the role of the funders for the present study and, if applicable, for the original study on which the present article is based<br>Page 25

*Give information separately for exposed and unexposed groups.

Note: An Explanation and Elaboration article discusses each checklist item and gives methodological background and published examples of transparent reporting. The STROBE checklist is best used in conjunction with this article (freely

For peer review only - http://bmjopen.bmj.com/site/about/guidelines.xhtml

available on the Web sites of PLoS Medicine at http://www.plosmedicine.org/, Annals of Internal Medicine at http://www.annals.org/, and Epidemiology at http://www.epidem.com/). Information on the STROBE Initiative is available at www.strobe-statement.org.

For peer review only

BMJ Open

The prevalence of mental health conditions, sensory impairments, and physical disability in people with co-occurring intellectual disabilities and autism compared with other people – a cross-sectional total population study in Scotland

Journal:	*BMJ Open*
Manuscript ID	bmjopen-2019-035280.R2
Article Type:	Original research
Date Submitted by the Author:	10-Mar-2020
Complete List of Authors:	Dunn, Kirsty; University of Glasgow, Health and Wellbeing<br>Rydzewska, Ewelina; University of Glasgow, Institute of Health and Wellbeing<br>Fleming, Michael ; University of Glasgow, Public Health<br>Cooper, Sally-Ann; Glasgow University , Institute of Health and Wellbeing
**Primary Subject Heading**:	Mental health
Secondary Subject Heading:	Public health
Keywords:	MENTAL HEALTH, Adult psychiatry < PSYCHIATRY, PUBLIC HEALTH

SCHOLARONE™
Manuscripts

BMJ

I, the Submitting Author has the right to grant and does grant on behalf of all authors of the Work (as defined in the below author licence), an exclusive licence and/or a non-exclusive licence for contributions from authors who are: i) UK Crown employees; ii) where BMJ has agreed a CC-BY licence shall apply, and/or iii) in accordance with the terms applicable for US Federal Government officers or employees acting as part of their official duties; on a worldwide, perpetual, irrevocable, royalty-free basis to BMJ Publishing Group Ltd ("BMJ") its licensees and where the relevant Journal is co-owned by BMJ to the co-owners of the Journal, to publish the Work in this journal and any other BMJ products and to exploit all rights, as set out in our licence.

The Submitting Author accepts and understands that any supply made under these terms is made by BMJ to the Submitting Author unless you are acting as an employee on behalf of your employer or a postgraduate student of an affiliated institution which is paying any applicable article publishing charge ("APC") for Open Access articles. Where the Submitting Author wishes to make the Work available on an Open Access basis (and intends to pay the relevant APC), the terms of reuse of such Open Access shall be governed by a Creative Commons licence – details of these licences and which Creative Commons licence will apply to this Work are set out in our licence referred to above.

Other than as permitted in any relevant BMJ Author's Self Archiving Policies, I confirm this Work has not been accepted for publication elsewhere, is not being considered for publication elsewhere and does not duplicate material already published. I confirm all authors consent to publication of this Work and authorise the granting of this licence.

For peer review only

The prevalence of mental health conditions, sensory impairments, and physical disability in people with co-occurring intellectual disabilities and autism compared with other people – a cross sectional total population study in Scotland

Kirsty Dunn*

Ewelina Rydzewska (University of Glasgow, Glasgow, UK)

Michael Fleming (University of Glasgow, Glasgow, UK)

Sally-Ann Cooper (University of Glasgow, Glasgow, UK)

*Corresponding author

Kirsty Dunn

Kirsty.wright@glasgow.ac.uk

07975867751

Institute of Health and Wellbeing

University of Glasgow

Glasgow

UK

For peer review only

Word count: 3,210

Keywords: Intellectual disabilities, autism, mental health conditions, sensory impairments, physical disability.

For peer review only

For peer review only - http://bmjopen.bmj.com/site/about/guidelines.xhtml

Abstract (281 words)

Objectives: To investigate prevalence of mental health conditions, sensory impairments, and physical disability in children, adults, and older adults with co-occurring intellectual disabilities and autism, given its frequent co-occurrence, compared with the general population.

Design: Whole country cohort study.

Setting: General community.

Participants: 5,709 people with co-occurring intellectual disabilities and autism, compared with 5,289,694 other people.

Outcome measures: Rates and odds ratios (OR) with 95% confidence intervals (95% CI) for mental health conditions, visual impairment, hearing impairment and physical disability in people with co-occurring intellectual disabilities and autism compared with other people, adjusted for age, sex, and interaction between age and co-occurring intellectual disabilities and autism.

Results: All four long-term conditions were markedly more common in children, adults, and older adults with co-occurring intellectual disabilities and autism compared with other people. For mental health, OR=130.8 (95% CI 117.1, 146.1); visual impairment OR=65.9 (95% CI 58.7, 73.9); hearing impairment OR=22.0 (95% CI 19.2, 25.2); physical disability OR=157.5 (95% CI 144.6, 171.7). These ratios are also greater than previously reported for people with *either* intellectual disabilities *or* autism rather than co-occurring intellectual disabilities and autism.

Conclusions: We have quantified the more than double disadvantage for people with co-occurring intellectual disabilities and autism, in terms of additional long-term health conditions. This may well impact on quality of life. It raises challenges for staff working with these people in view of additional

For peer review only - http://bmjopen.bmj.com/site/about/guidelines.xhtml

complexity in assessments, diagnoses, and interventions of additional health conditions, as sensory impairments and mental health conditions in particular, compound with the persons pre-existing communication and cognitive problems in this context. Planning is important, with staff being trained, equipped, resourced and prepared to address the challenge of working for people with these conditions.

Strengths and limitations of this study

- Large scale, whole country study, with a high response rate (94%), so the results are representative of the whole population.
- Intellectual disabilities, autism, and additional long-term conditions were enquired about systematically for everyone in the population.
- The wording of questions was tested in advance, via cognitive question testing during the design of Scotland's Census, 2011.
- Limitations include proxy-reporting.
- People known to have autism/Asperger's syndrome, intellectual disabilities, and the four long-term conditions were reported, rather than each undergoing detailed individual research assessments which are not possible in such large population studies.

For peer review only

Background

People with intellectual disabilities[1-4] and people with autism[5-7] have more mental and physical health needs than other people. A whole population study using the Scotland Census 2011 reported that 21.7% of people with intellectual disabilities also had autism, and 18.0% of people with autism also had intellectual disabilities,[8] so this dually diagnosed group warrant investigation. One would suspect that this population with co-occurring intellectual disabilities and autism is likely to have a high level of additional health needs, but this has received little previous attention. A higher number of additional health needs increases the likelihood of misdiagnosis, and treatment interactions, so requires more complex treatment plans. Hence it is important to investigate long-term additional health needs experienced by people with co-occurring intellectual disabilities and autism.

Some studies have investigated mental ill-health in people with co-occurring intellectual disabilities and autism. A small study of 149 adults with severe or profound intellectual disabilities and autism, living in state-run developmental centres in Louisiana, USA, compared co-existing conditions with 158 adults with intellectual disabilities without autism in the same centres. The former group had more symptomology for anxiety, mania, schizophrenia, stereotypies, self-injurious behaviour, eating disorders, sexual disorders, and impulse control.[9] A study in Norway compared 62 adults with co-occurring autism and intellectual disabilities under the care of autism services, with 132 adults with intellectual disabilities only receiving intellectual disabilities support.[10] High levels of psychiatric disorders were reported in both groups; 53.2% in the co-occurring intellectual disabilities and autism group, and 17.4%

For peer review only - http://bmjopen.bmj.com/site/about/guidelines.xhtml

in the intellectual disabilities only group. An English study of referrals to a specialist intellectual disabilities psychiatric service described 42% of the 137 referred adults who had autism as well as intellectual disabilities to have co-existing psychopathology, most commonly schizophrenia.[11] A study of youth aged 14-20 years age, gender matched 36 people with co-occurring intellectual disabilities and autism with 36 people with intellectual disabilities without autism.[12] They reported the former group to have more episodes of mental ill-health, most commonly depression. A study of people aged 8-29 years with intellectual disabilities and challenging behaviour living in four residential units in England included 69 who also had autism and 13 who did not.[13] They reported a higher prevalence of organic disorders, anxiety and stereotypies in the young people with co-occurring intellectual disabilities and autism. This literature is difficult to summarise overall, as, as well as having small sample sizes, the participants were not drawn from representative populations.

A further study had the advantage of being population-based, but was still small in size.[14] It compared the prevalence, and incidence, of mental ill-health in 77 adults with co-occurring intellectual disabilities and autism with 946 adults with intellectual disabilities without autism, and also with 154 individually age, gender, ability-level, and Down syndrome matched controls. The adults with autism had a higher point prevalence of problem behaviours than the 946 without autism, but compared with the 154 matched controls there was no difference in prevalence, or incidence of either problem behaviours or other mental ill-health.[14] Three large whole population studies using the Scotland Census 2011 have reported that of people with intellectual disabilities, 21.7% reported mental health conditions;[3] and of people with autism, 33.0% of adults,[6] and 7.6% of children[7] reported mental health

For peer review only - http://bmjopen.bmj.com/site/about/guidelines.xhtml

conditions, but did not report the rates for people with co-occurring intellectual disabilities and autism.

A recent study of Medicare claims data in Wisconsin, USA, by Bishop & Rubenstein (2019) compared the physical and mental health conditions for adults aged 40-88 years old with a diagnosis of autism only (N= 79) to those with a diagnosis of both autism and intellectual disabilities (N= 64) between 2012 and 2015. The prevalence of chronic medical conditions was high among the entire sample, with elevated but not statistically significant prevalence rates for adults with both autism and intellectual disabilities on most conditions. However, odds ratios revealed a decreased likelihood of anxiety and depression for individuals with both autism and intellectual disabilities, and a higher likelihood of epilepsy compared to those with autism only[15]. As the adults in this study were registered with Medicare, the sample may represent a lower socioeconomic group than those with autism in the general population. Given the age of the sample, and the changes in diagnostic criteria for autism, this sample may not represent adults who are at the high functioning end of the spectrum.

With regards to sensory impairments, of the 36 matched youth with intellectual disabilities with and without autism, 38.9% with autism reported having visual problems compared with 50.0% without autism, and 13.9% with autism reported having hearing problems compared with 19.4% without autism.[12] An intellectual disabilities register study reported that 95 of the 368 (25.8%) adults with intellectual disabilities who had visual impairment also had markers for autism, compared with 422 of 2,674 (16%) of those who had normal vision, and that 46 of the 60 (76.7%) of the adults with intellectual

For peer review only - http://bmjopen.bmj.com/site/about/guidelines.xhtml

disabilities and congenital blindness also had markers for autism, compared with only 36 of the 67 (53.7%) with normal vision.[16]

We have not identified other papers on sensory impairments or any on physical disabilities in people with co-occurring intellectual disabilities and autism. However, previous large whole population studies which analysed data from the Scotland Census 2011 have reported that of people with intellectual disabilities, 12.4% reported blindness/sight loss, 13.1% reported deafness/hearing loss, and 32.6% reported physical disability.[3] Of people with autism, 12.1% of adults[6] and 3.5% of children reported blindness/sight loss,[7] 14.1% of adults[6] and 2.9% of children reported deafness/hearing loss,[,7] and 24.0% of adults[6] and 10.7% of children reported physical disability.[7] They did not, however, report the rates of these conditions for people with co-occurring intellectual disabilities and autism.

This high prevalence of health conditions among people with intellectual disabilities and people with autism spectrum disorders is partly attributable health inequalities[17,18], and to the fact that certain conditions, such as cerebral palsy, are associated with both intellectual and physical disabilities. Additionally, the socioeconomic status of individuals within these populations is typically lower than for members of the general population[19,20]. While prevalence rates of health conditions in a full country population for individuals with intellectual disabilities and individuals with autism have been compared to the general population in previous work using the Scotland Census 2011[3,4,7], no such study has been conducted on the prevalence of health conditions for those with both intellectual disabilities and autism.

For peer review only - http://bmjopen.bmj.com/site/about/guidelines.xhtml

The management of care and treatment plans for individuals with multiple health conditions or disabilities presents significant challenges for health care practitioners. A review of 123 studies on care management for individuals with multiple chronic conditions in the USA reported that these patients access services more frequently and use a larger range of services than other patients, making the coordination of their care more difficult and often leading to suboptimal care[21]. Existing evidence also suggests that when individuals with intellectual disabilities have additional long-term conditions, these conditions are more poorly managed than for members of the general population with the same conditions[22].

Given the frequent overlap of intellectual disabilities and autism, information on the associated co-existing conditions is important, to assist policy makers, planners, and practitioners to best adapt services for individuals with co-occurring intellectual disabilities and autism. This paper aims to investigate the prevalence of mental health conditions, sensory impairments, and physical disability in children, adults, and older adults with co-occurring intellectual disabilities and autism, compared with other people.

Methods

Approval

Approval was obtained from the Scottish Government to undertake secondary data analysis of Scotland's Census, 2011.

For peer review only - http://bmjopen.bmj.com/site/about/guidelines.xhtml

Data Source

Scotland's Census provides information on Scotland's population every ten years, with the most recent Census on 27th March 2011.[23] The Census provides information on the number and characteristics of S11cotland's population and households on the Census date.

It is a legal requirement to complete the census form and households were informed that failure to make a Census return, or supplying false information could result in a £1,000 fine. A very high response rate was achieved, with an estimated 94% of all of Scotland's population completing the Census. The Census team used a Census Coverage Survey with about 40,000 households, to estimate numbers and characteristics of the missing 6%.[24] The Coverage Survey and Census records were deterministically matched to check for duplicates. Individuals estimated to have been missed were then imputed using a subset of characteristics from real individuals. The edit and imputation methodology was adapted from the Office for National Statistics rigorous and systematic guidelines.[24]

The Census was completed by the head of each household on behalf of all occupants of the household. We consider it unlikely that people with co-occurring intellectual disabilities and autism completed the form, given the reading age required to do so. Rather, we expect that the people who completed the form on their behalf were parent-carers in family households, support workers for people living in supported accommodation, and the managers/key workers at communal establishments.

For peer review only - http://bmjopen.bmj.com/site/about/guidelines.xhtml

Variables

The census included a question to identify people with intellectual disabilities and autism, as well as mental health conditions, sensory impairments, and physical disabilities: 'Do you have any of the following conditions which have lasted, or are expected to last, at least 12 months? Tick all that apply'. There was a choice of ten possible responses to this question: deafness or partial hearing loss; blindness or partial sight loss; learning disability (for example, Down's syndrome); learning difficulty (for example, dyslexia); developmental disorder (for example, Autistic Spectrum Disorder or Asperger's Syndrome); physical disability; mental health condition; long-term illness, disease or condition; other condition. For "other condition" the option of providing more detail in an open text response was provided.

In Scotland, the term "learning disability" is used synonymously with that of "intellectual disabilities" used internationally. Importantly, the Census differentiated between intellectual disabilities and specific learning disabilities; and between intellectual disabilities and autism.

During the methodology development for Scotland's Census, 2011, cognitive question testing was undertaken on the questions on long-term health conditions and disabilities. This was to determine whether the questions were answered accurately, and to identify any changes needed to improve data quality and/or the acceptability of the way questions were phrased. Cognitive interviewing is a widely used approach to critically evaluate and improve survey questionnaires.[25] This approach enables researchers to modify survey material to enhance clarity. Retrospective probing was conducted with 102 participants with a variety of sex, age, and health conditions and disabilities

For peer review only - http://bmjopen.bmj.com/site/about/guidelines.xhtml

(including people with more than one of the conditions). They included people with autism, intellectual disabilities, dyslexia, dyspraxia, speech impairment, mental health conditions (both milder and more serious), and other long-term conditions.[23] Using the cognitive interviewing results, the question aimed to detect autism was improved and rephrased, to better capture this information. The questions on intellectual disability, mental health condition, visual impairment, hearing impairment, and physical disability did not require any modifications[26].

The Census team imputed answers for the 14.7% who did not tick any of the boxes in question on long-term conditions, based on their free text answers for this question and answers to other health questions in the Census, which increased the completion rate to 97.4%. For the remaining 2.6%, the Census team assumed the most plausible explanation was that the person had no long-term condition but did not see the "No condition" check box at the end of the question, and hence recorded them as having no conditions.

Data Analysis

We calculated the number and rate per 1,000 population of children and adults with co-occurring intellectual disabilities and autism. We then calculated the number and percentage of people with mental health conditions, deafness or partial hearing loss, blindness or partial sight loss, and physical disability, for those with co-occurring intellectual disabilities and autism, compared with individuals who do not have co-occurring intellectual disabilities and autism using chi-squared (χ^2) tests. For the whole population we then used logistic regression to calculate the odds ratios (OR: 95% confidence interval, 95% CI) of

For peer review only - http://bmjopen.bmj.com/site/about/guidelines.xhtml

co-occurring intellectual disabilities and autism statistically predicting the binary response of having each of the four specific types of long-term health conditions, adjusted for age and sex. Sex was binary, with males being the reference group. Age was categorised into groups: 0-15, 16-24, 25-34, 35-44, 45-54, 55-64, 65-74, 75+, with 0-15 years as the reference group. We repeated the regressions, including the interaction term of age x co-occurring intellectual disabilities and autism, as people with the most severe disabilities die earlier, which may affect the profile of additional health problems differently to that seen in the general population. The same reference groups were used. All analysis was conducted using SPSS software version 22.

For peer review only

Patient and Public Involvement

The Scottish Learning Disabilities Observatory, where this research was undertaken, has a specific remit for people with intellectual disabilities and people with autism. Its steering group includes partners from third sector organisations and experts by experience, who approved the workplan for this project prior to it commencing. Results from this study will be disseminated for people with intellectual disabilities and autism in an easy-read version via the Scottish Learning Disabilities Observatory website, newsletters, and conference.

Results

Characteristics of the Sample

Scotland's Census, 2011, includes records on 5,295,403 people aged 0-75+ years. 5,709/5,295,403 (1.08/1,000) people had co-occurring intellectual disabilities and autism; of whom 3,769 (66.0%) were male and 1,940 (44.0%)

For peer review only - http://bmjopen.bmj.com/site/about/guidelines.xhtml

were female. Overall, 2,362/916,331 (2.58/1,000) of the total population of children (0-15 years), and 3,347/4,379,072 (0.76/1,000) adults (16-75+ years) had co-occurring intellectual disabilities and autism.

Compared with the population who did not have co-occurring intellectual disabilities and autism, the population with co-occurring intellectual disabilities and autism had more males (66.0% versus 48.5%; χ^2=703.5; df=1; p<0.001); were younger (χ^2=3894.7; df=7; p<0.001); were more likely to have been born in the UK rather than elsewhere (χ^2=101.9; df=1; p<0.001), revealing lesser geographic mobility; and were no different with regards to Caucasian versus non-Caucasian ethnicity (χ^2=1.1; df=1; p=0.3) (table 1).

- Insert table 1 about here -

Long-term Health Conditions

Table 2 shows the proportion of people with co-occurring intellectual disabilities and autism, who had each of the four additional long-term health conditions, compared to people who did not have co-occurring intellectual disabilities and autism. Some people in the sample had more than one long-term health condition.

- Insert table 2 about here -

Mental Health Condition

Adjusting for age and sex, given the different distributions compared with the general population, having co-occurring intellectual disabilities and autism had an OR=25.55 (23.93-27.28, 95% CI) in predicting mental health conditions

For peer review only - http://bmjopen.bmj.com/site/about/guidelines.xhtml

(table 3). When the interaction term was added (age x co-occurring intellectual disabilities and autism), co-occurring intellectual disabilities and autism had an OR=130.80 (117.13-146.07, 95% CI) in predicting a mental health condition (table 3).

- Insert table 3 about here -

Blindness or partial sight loss

Adjusting for age and sex, having co-occurring intellectual disabilities and autism had an OR=36.78 (34.21-39.54, 95% CI) in predicting blindness or partial sight loss (table 4). When the interaction term was added (age x co-occurring intellectual disabilities and autism), co-occurring intellectual disabilities and autism had an OR=65.90 (58.74-73.92, 95% CI) in predicting blindness or partial sight loss (table 4).

- Insert table 4 about here -

Deafness or partial hearing loss

Adjusting for age and sex, having co-occurring intellectual disabilities and autism had an OR=11.33 (10.43-12.31, 95% CI) in predicting deafness or partial hearing loss (table 5). When the interaction term was added (age x co-occurring intellectual disabilities and autism), co-occurring intellectual disabilities and autism had an OR=22.00 (19.20-25.21, 95% CI) in predicting deafness or partial hearing loss (table 5).

- Insert table 5 about here -

For peer review only - http://bmjopen.bmj.com/site/about/guidelines.xhtml

For peer review only

Physical disability

Adjusting for age and sex, having co-occurring intellectual disabilities and autism had an OR=61.16 (57.60-64.94, 95% CI) in predicting physical disability (table 6). When the interaction term was added (age x co-occurring intellectual disabilities and autism), co-occurring intellectual disabilities and autism had an OR=157.54 (144.58-171.66, 95% CI) in predicting physical disability (table 6).

- Insert table 6 about here -

Discussion

Principle findings

Mental health conditions, blindness or partial sight loss, deafness or partial hearing loss, and physical disability were all significantly more common in people with co-occurring intellectual disabilities and autism than people without these co-occurring conditions. The odds ratios after adjusting for age and sex and the interaction term, were substantial, being 131, 66, 22, and 158 respectively. This is important as each of these conditions are disabling and can significantly impact an individual's quality of life. They contribute to high rates of multi-morbidity, which, on top of communication and cognitive problems due to autism and intellectual disabilities, renders assessments, diagnosis, and treatment of additional health problems more complex than for other people.

Across all age groups, blindness, deafness, and physical disability were more common in females then males with co-occurring intellectual disabilities and autism, unlike the gender ratios in people without co-occurring intellectual disabilities and autism. Mental health conditions were more common in males

For peer review only - http://bmjopen.bmj.com/site/about/guidelines.xhtml

than females with co-occurring intellectual disabilities and autism, except for the 65+ year group, contrary to the gender ratios in other people. All conditions were more prevalent with increasing age in the people with co-occurring intellectual disabilities and autism, except for physical disability which was more common in the children/youth and older people than in the adults.

Comparison with existing literature

The prevalence of these additional long-term health conditions has seldom been investigated in people with co-occurring intellectual disabilities and autism, particularly in comparison with other people, and never, to our knowledge, as a total population study. All of the long-term health conditions were more common than in those without co-occurring intellectual disabilities and autism.

Smaller, less representative studies have reported a higher rate of mental health conditions in adults and youth with co-occurring intellectual disabilities and autism compared with those with intellectual disabilities and without autism,[9-13] but not all.[14] People with autism have been reported to have more mental health conditions than other people (OR=9 in adults and OR=16 in children),[6,7] as have people with intellectual disabilities compared with other people (OR=7),[3] using the same Scotland's Census, 2011 data as in this current paper, whereas the comparable ratio we now report for people with co-occurring intellectual disabilities and autism for mental health conditions was OR=26. Having the co-occurring conditions therefore presents a much higher risk of mental health conditions than either intellectual disabilities or autism on their own.

For peer review only - http://bmjopen.bmj.com/site/about/guidelines.xhtml

The previous small study of youth reported lower rates of visual and hearing impairments in those with co-occurring intellectual disabilities and autism (38.9% and 13.9%) compared with those with intellectual disabilities but without autism (50% and 19.4%).[12] This was in contrast with the larger study reporting more autistic symptoms in adults with intellectual disabilities and visual impairments than in adults with intellectual disabilities but without visual impairments.[16] Adults with autism have been reported to have more blindness or partial sight loss, and deafness or partial hearing loss than other people (12.1% and 17.5%),[6] as have children with autism (3.5% and 2.9%),[7] and people (children and adults combined) with intellectual disabilities compared with other people (13.1% and 12.4%),[3] using the same data from Scotland's Census 2011 as in this current paper. This current study found the comparable rates for people with co-occurring intellectual disabilities and autism for blindness or partial sight loss, and deafness or partial hearing loss was 21.7% and 19.3% for adults, and 16.6% and 10.3% for children. Having the co-occurring conditions of intellectual disabilities and autism therefore presents a much higher risk of sensory impairments than for children and adults with autism, and for people with intellectual disabilities (although children were not separately studied in the previous report).

Regarding physical disability, 32.6% of people with intellectual disabilities were previously reported to have physical disability using the same dataset as the current study.[3] Of people with autism, 24.0% of adults and 10.7% of children reported physical disability in this dataset.[6,7] These rates are lower than those we report in this current study of people with co-occurring intellectual disabilities and autism – 45.6% of children and 42.2% of adults.

For peer review only - http://bmjopen.bmj.com/site/about/guidelines.xhtml

Strengths and limitations

Strengths of the study include its large scale and general population comparison group, whole population coverage and very high response rate so the results are representative of the whole population. Intellectual disabilities, autism, and the long-term health conditions were enquired about systematically for everyone in the population. We believe the results are therefore generalisable to other high-income countries. The cognitive question testing during the design of the Census is a further strength. The Census had clear categories to distinguish between intellectual disabilities, specific learning disability (like dyslexia), and autism.

Limitations include the proxy-reporting, which may, or may not reflect self-reports. However, without proxy-reports, we would have no information on people unable to self-report due to their disabilities, and a previous review on the topic concluded that overall, proxy reports are a useful addition to determine aspects of well-being in people with intellectual disabilities.[27] Additionally, people were reported who were known to have autism/Asperger's syndrome, intellectual disabilities, and the additional long-term health conditions, rather than detailed individual research assessments being undertaken which are clearly not possible in such large population studies, and may therefore be subject to a degree of error which we were not able to check. Individuals who were known to have intellectual disabilities and autism are higher health care users and so are more likely to receive a diagnosis for other health care conditions than members of the general population who do not access health care services as frequently. It is also important to note that the Scotland Census 2011 was administered 8 years

For peer review only - http://bmjopen.bmj.com/site/about/guidelines.xhtml

ago, and so any potential changes in prevalence rates of the conditions investigated in this paper are not captured by this analysis.

Implications

There is a greater than double disadvantage for people with co-occurring intellectual disabilities and autism, in terms of additional long-term health conditions. We found that, and quantified the extent to which mental health conditions, sensory impairments, and physical disabilities are more common than in people without co-occurring intellectual disabilities and autism, and in people with just intellectual disabilities or just autism. This may well impact on quality of life. It raises challenges for staff working with people with these co-occurring conditions in view of the additional complexity in assessments, diagnoses, and interventions, as sensory impairments and mental health conditions in particular, interact with the persons pre-existing communication and cognitive problems in this context. Therefore, it is important that these co-occurring conditions are planned for with staff being trained, equipped, resourced and prepared to address the challenge.

References

1. Cooper S-A, McLean G, Guthrie B, McConnachie A, Mercer S, Sullivan F, Morrison. J. Multiple physical and mental health comorbidity in adults with intellectual disabilities: population-based cross-sectional analysis. *BMC Family Practice* 2015; 16: 110.
2. Hughes-McCormack LA, Rydzewska E, Henderson A., Rintoul J, Macintyre C, Cooper S-A. Prevalence and general health status of people with intellectual disabilities in Scotland -a total population study. *J Epidemiol Community Health 2018;* 72: 78–85.

For peer review only - http://bmjopen.bmj.com/site/about/guidelines.xhtml

3. Hughes-McCormack LA, Rydzewska E, Henderson A, Rintoul J, Macintyre C, Cooper S-A. Prevalence of mental ill-health and relationship with physical health in a whole country population of 26,349 people with intellectual disabilities, compared with the general population. *Br J Psychiatry Open,* 2017*;* 3: 243-248.
4. Kinnear D, Morrison J, Allan L, Henderson A, Smiley E, & Cooper S-A. Prevalence of physical conditions and multimorbidity in a cohort of adults with intellectual disabilities with and without Down syndrome: cross-sectional study. *BMJ Open* 2018; *8*:e018292.
5. Croen LA, Zerbo O, Qian Y, Massolo ML, Rich S, Sidney S, Kripke C. The health status of adults on the autism spectrum. *Autism* 2015; 19:814–823.
6. Rydzewska E, Hughes-McCormack LA, Gillberg C, Henderson A, MacIntyre C, Rintoul J, & Cooper S-A. Prevalence of long-term health conditions in adults with autism: observational study of a whole country population. *BMJ Open* 2018a; 8(8): e023945.
7. Rydzewska E, Hughes-McCormack LA, Gillberg C, Henderson A, MacIntyre C, Rintoul J, Cooper S-A. Prevalence of sensory impairments, physical and intellectual disabilities, and mental health in children and young people with self/proxy-reported autism - observational study of a whole country population. *Autism* 2018; 23:1201-9.
8. Dunn K, Rydzewska E, Macintyre C, Rintoul J, & Cooper S-A. The prevalence and general health status of people with intellectual disabilities and autism co-occurring together: a total population study. *J Inellectual Disabilit Res* 2018, 63: 277-285.
9. Cervantes PE, Matson JL. Comorbid symptomology in adults with autism spectrum disorder and intellectual disability. *J Autism Dev Disord* 2015; *45(12)*: 3961-3970.

For peer review only

For peer review only - http://bmjopen.bmj.com/site/about/guidelines.xhtml

10. Bakken TL, Helverschou SB, Eilertsen DE, Heggelund T, Myrbakk E, & Martinsen, H. Psychiatric disorders in adolescents and adults with autism and intellectual disability: A representative study in one county in Norway. *Res Dev Disabil* 2010; *31*: 1669-1677.

11. Tsakanikos E, Sturmey P, Costello H, Holt G, Bouras N. Referral trends in mental health services for adults with intellectual disability and autism spectrum disorders. *Autism* 2007;11:9-17.

12. Bradley E, Bolton P. Episodic psychiatric disorders in teenagers with learning disabilities with and without autism. *Br J Psychiatr* 2006; 189:361-6.

13. Hill J, Furniss F. Patterns of emotional and behavioural disturbance associated with autistic traits in young people with severe intellectual disabilities and challenging behaviours. *Res Dev Disabil* 2006; 27:517-28.

14. Melville CA, Cooper S-A, Morrison J, Smiley E, Allan L, Jackson A, Finlayson J, Mantry D. The prevalence and incidence of mental ill-health in adults with autism and intellectual disabilities. *J Autism Dev Disord* 2008; 38:1676-88.

15. Bishop-Fitzpatrick L, Rubenstein E. The physical and mental health of middle aged and older adults on the autism spectrum and the impact of intellectual disability. Research in Autism Spectrum Disorders. 2019b Jul 1; 63:34-41.

16. Kiani, R, Bhaumik, S, Tyrer, F, Bankart, J, Miller, H, Cooper, S-A, Brugha, T. The relationship between autism spectrum disorder and visual impairments among adults with intellectual disability. *Autism Res 2019;* 12:1411-1422.

17. Evenhuis, H., Henderson, C.M., Beange, H., Lennox, N. and Chicoine, B., 2001. Healthy ageing–adults with intellectual disabilities: physical health issues. Journal of Applied Research in Intellectual Disabilities, 14(3), pp.175-194.

18. Heslop, P., Blair, P. S., Fleming, P., Hoghton, M., Marriott, A., & Russ, L. 2014. The Confidential Inquiry into premature deaths of people with

For peer review only

intellectual disabilities in the UK: a population-based study. The Lancet, 383 (9920), pp. 889-895.

19. Shifrer D, Muller C, Callahan R. Disproportionality and learning disabilities: Parsing apart race, socioeconomic status, and language. Journal of learning disabilities. 2011 May;44(3):246-57.
20. Emerson E. Poverty and people with intellectual disabilities. Mental retardation and developmental disabilities research reviews. 2007;13(2):107-13.
21. Vogeli C, Shields AE, Lee TA, Gibson TB, Marder WD, Weiss KB, Blumenthal D. Multiple chronic conditions: prevalence, health consequences, and implications for quality, care management, and costs. Journal of general internal medicine. 2007 Dec 1;22(3):391-5.
22. Cooper SA, Hughes-McCormack L, Greenlaw N, McConnachie A, Allan L, Baltzer M, McArthur L, Henderson A, Melville C, McSkimming P, Morrison J. Management and prevalence of long-term conditions in primary health care for adults with intellectual disabilities compared with the general population: A population-based cohort study. Journal of Applied Research in Intellectual Disabilities. 2018 Jan;31:68-81.
23. National Records of Scotland. Scotland's Census, 2011. Supporting information. *National Records of Scotland*. 2011 http://www.scotlandscensus.gov.uk/supporting-information.
24. Office for National Statistics. Survey Methodology Bulletin No. 69 – September 2011. 2011 https:// webarchive.nationalarchives.gov.uk/20160108193745/http://www. ons.gov.uk/guide-method/method-quality/survey-methodologybulletin/smb-69/index.html.

For peer review only

25. Willis GB. Cognitive interviewing: A tool for improving questionnaire design. Sage Publications; 2004.
26. General Register Office for Scotland. 2011 Census Question Testing- The Health and Disability Questions (2009). 2009 https://www.scotlandscensus.gov.uk/documents/research/2011-census-healthdisability-questions.pdf.
27. Perkins EA, Berkman KA. Into the unknown: Aging with autism spectrum disorders. *Am J Intellect Dev Disabil* 2012; 117:478-96.

Funding

This work was supported by the UK Medical Research Council, grant number: MC_PC_1717), and the Scottish Government via the Scottish Learning Disabilities Observatory.

The study sponsor and funders had no role in the study design; in the collection, analysis, and interpretation of data; in the writing of the report; and in the decision to submit the article for publication.

The researchers are independent from the funders.

Competing interests

The authors declare no competing interests.

Author's contributions

For peer review only - http://bmjopen.bmj.com/site/about/guidelines.xhtml

KD analysed and interpreted the data, and wrote the first draft of the manuscript. ER contributed to data access, data interpretation, and drafting the manuscript. MF provided advice on response to reviewer comments regarding choice of statistical methods and contributed to the writing of the manuscript. S-AC conceived and managed the project, interpreted data, and contributed to writing the manuscript. All approved the final version of the manuscript. S-AC is the study guarantor.

Data sharing

Data is available via National Records of Scotland, following project approval. Data are available at the following link https://www.scotlandscensus.gov.uk/ods-web/data-warehouse.html#additionaltab

Acknowledgments

We thank the National Records of Scotland Census team, and the Scottish population who completed the Census.

For peer review only - http://bmjopen.bmj.com/site/about/guidelines.xhtml

Table 1: Characteristics of people with and without, co-occurring intellectual disabilities and autism

	**People without co-occurring intellectual disabilities and autism**<br>N=5,289,694 (100%)<br>Number (%)	**People with co-occurring intellectual disabilities and autism**<br>N=5,709 (100%)<br>Number (%)
**Gender***		
Males	2,563,675 (48.5%)	3,769 (66.0%)
Females	2,726,019 (51.5%)	1,940 (44.0%)
**Age groups***		
0-15	913,969 (17.3%)	2,362 (41.4%)
16-24	631,094 (11.9%)	1,394 (24.4%)
25-34	666,725 (12.6%)	602 (10.5%)
35-44	734,304 (13.9%)	450 (7.9%)
45-54	786,355 (14.9%)	401 (7.0%)
55-64	667,157 (12.6%)	256 (4.5%)
65+	890,090 (16.8%)	244 (4.3%)
**Country of birth***		
UK	4,920,614 (93.0%)	5,505 (96.4%)
Other Europe	172,160 (3.3%)	83 (1.5%)
Africa	46,708 (0.9%)	34 (0.6%)
Middle East and Asia	104,480 (2.0%)	50 (0.9%)
The Americas and the Caribbean	33,325 (0.6%)	28 (0.5%)
Other	12,407 (0.2%)	9 (0.2%)
**Ethnicity**		
White	5,078,910 (96.0%)	5,497 (96.3%)
Asian	140,542 (2.7%)	136 (2.4%)
Mixed/multiple ethnicities	19,775 (0.4%)	40 (0.7%)

For peer review only - http://bmjopen.bmj.com/site/about/guidelines.xhtml

African	29,615 (0.6%)	23 (0.4%)
Caribbean or black	6,536 (0.1%)	4 (0.1%)
Other ethnic groups	14,316 (0.3%)	9 (0.2%)

*People with co-occurring intellectual disabilities and autism versus people without co-occurring intellectual disabilities and autism; $p<0.01$

For peer review only

Table 2: Prevalence of conditions in people with and without co-occurring intellectual disabilities and autism by age and sex

People with co-occurring intellectual disabilities and autism									
**Condition**	**Children/youth, 0-15 years** N=2,362			**Adults, 16-64 years** N=3,103			**Older people, 65+ years** N=244		
	**M** N=1,563 (100%)	**F** N=799 (100%)	**Total** N=2,362 (100%)	**M** N= 2,073 (100%)	**F** N= 1,030 (100%)	**Total** N= 3,103 (100%)	**M** N= 133 (100%)	**F** N= 111 (100%)	**Total** N= 244 (100%)
**Mental health condition**	328 (21.0%)	152 (19.0%)	480 (20.3%)	768 (37.0%)	377 (36.6%)	1145 (36.9%)	80 (60.2%)	80 (72.1%)	160 (65.6%)
**Blindness/partial sight loss**	214 (13.7%)	177 (22.2%)	391 (16.6%)	355 (17.1%)	220 (21.4%)	575 (18.5%)	71 (53.3%)	80 (72.1%)	151 (61.9%)
**Deafness/partial hearing loss**	148 (9.5%)	95 (11.9%)	243 (10.3%)	301 (14.5%)	190 (18.4%)	491 (15.8%)	73 (54.9%)	81 (73.0%)	154 (63.1%)
**Physical disability**	618 (39.5%)	458 (57.3%)	1,076 (45.6%)	719 (34.7%)	508 (49.3%)	1,227 (39.5%)	86 (64.7%)	99 (89.2%)	185 (75.8%)

People without co-occurring intellectual disabilities and autism									
**Condition**	**Children/youth, 0-15 years** N=913,969			**Adults, 16-64 years** N=3,485,635			**Older people, 65+ years** N=890,090		
	**M** N=467,543 (100%)	**F** N=446,426 (100%)	**Total** N= 913,969 (100%)	**M** N= 1,712,526 (100%)	**F** N= 1,773,109 (100%)	**Total** N= 3,485,635 (100%)	**M** N= 383,606 (100%)	**F** N= 506,484 (100%)	**Total** N= 890,090 (100%)
**Mental health condition**	1,861 (0.4%)	980 (0.2%)	2,841 (0.3%)	92,308 (5.4%)	95,108 (5.4%)	187,416 (5.4%)	14,760 (3.8%)	26,141 (5.2%)	40,901 (4.6%)
**Blindness/partial sight loss**	1,793 (0.4%)	1,439 (0.3%)	3,232 (0.4%)	24,129 (1.4%)	16,954 (1.0%)	41,083 (1.2%)	30,389 (7.9%)	49,839 (9.8%)	80,228 (9.0%)
**Deafness/partial hearing loss**	2,731 (0.6%)	2,225 (0.5%)	4,956 (0.5%)	70,543 (4.1%)	48,727 (2.7%)	119,270 (3.4%)	111,447 (29.1%)	114,393 (22.6%)	225,840 (25.4%)
**Physical disability**	3,637 (0.8%)	2,799 (0.6%)	6,436 (0.7%)	81,655 (4.8%)	82,968 (4.7%)	164,623 (4.7%)	73,759 (19.2%)	109,103 (21.5%)	182,862 (20.5%)

For peer review only - http://bmjopen.bmj.com/site/about/guidelines.xhtml

Table 3: Independent predictors of mental health conditions in the whole population

**Characteristic**		**Regression 1**		**Regression 2 (including the interaction term: age x co-occurring intellectual disabilities and autism)**	
		**Odds ratio**	**95% confidence interval**	**Odds ratio**	**95% confidence interval**
Co-occurring intellectual disabilities and autism	Not present (reference)	-	-	-	-
	Co-occurring intellectual disabilities and autism	25.55	23.93-27.28	130.80	117.13-146.07
Gender	Male (reference)	-	-	-	-
	Female	1.28	1.26-1.29	1.28	1.26-1.29
Sex	0-15 (reference)	-	-	-	-
	16-24	9.45	9.04-9.88	11.32	10.78-11.89
	25-34	21.17	20.29-22.09	25.31	24.13-26.53
	35-44	29.73	28.50-31.00	35.49	33.87-37.20
	45-54	29.19	27.98-30.44	34.84	33.24-36.51
	55-64	22.63	21.63-23.62	27.00	25.75-28.30
	65+	19.320	18.52-20.16	23.00	21.95-24.12
Age x both intellectual disabilities and autism	0-15 (reference)	-	-	-	-
	16-24	-	-	0.15	0.13-0.18
	25-34	-	-	0.10	0.08-0.12
	35-44	-	-	0.08	0.07-0.10
	45-54	-	-	0.09	0.07-0.11
	55-64	-	-	0.12	0.09-0.15
	65+	-	-	0.31	0.24-0.42
Constant	-	0.00	-	0.00	-

For peer review only

For peer review only - http://bmjopen.bmj.com/site/about/guidelines.xhtml

Table 4: Independent predictors of blindness/partial sight loss in the whole population

Characteristic		Regression 1		Regression 2 (including the interaction term: age x co-occurring intellectual disabilities and autism)	
		**Odds ratio**	**95% confidence interval**	**Odds ratio**	**95% confidence interval**
Co-occurring intellectual disabilities and autism	Not present (reference)	-	-	-	-
	Co-occurring intellectual disabilities and autism	36.78	34.21-39.54	65.90	58.74-73.92
Sex	Male (reference)	-	-	-	-
	Female	1.01	1.00-1.02	1.01	1.00-1.02
Age	0-15 (reference)	-	-	-	-
	16-24	1.56	1.48-1.64	1.66	1.58-1.75
	25-34	1.82	1.74-1.91	1.91	1.82-2.01
	35-44	2.55	2.44-2.66	2.69	2.57-2.81
	45-54	4.42	4.24-4.60	4.67	4.48-4.87
	55-64	7.50	7.22-7.80	7.93	7.61-8.26
	65+	31.06	29.95-32.22	32.75	31.51-34.04
Age x both intellectual disabilities and autism	0-15 (reference)	-	-	-	-
	16-24	-	-	0.43	0.35-0.53
	25-34	-	-	0.67	0.52-0.84
	35-44	-	-	0.53	0.41-0.68
	45-54	-	-	0.37	0.29-0.47
	55-64	-	-	0.28	0.21-0.37
	65+	-	-	0.25	0.19-0.33
Constant	-	0.00	-	0.00	-

For peer review only - http://bmjopen.bmj.com/site/about/guidelines.xhtml

Table 5: Independent predictors of deafness/partial hearing loss in the whole population

Characteristic		Regression 1		Regression 2 (including the interaction term: age x co-occurring intellectual disabilities and autism)	
		**Odds ratio**	**95% confidence interval**	**Odds ratio**	**95% confidence interval**
Co-occurring intellectual disabilities and autism	Not present (reference)	-	-	-	-
	Co-occurring intellectual disabilities and autism	11.33	10.43-12.31	22.00	19.20-25.21
Gender	Male (reference)	-	-	-	-
	Female	0.69	0.68-0.69	0.69	0.68-0.69
Sex	0-15 (reference)	-	-	-	-
	16-24	1.56	1.50-1.62	1.59	1.52-1.65
	25-34	2.36	2.27-2.45	2.41	2.32-2.50
	35-44	4.24	4.10-4.38	4.35	4.20-4.50
	45-54	8.55	8.29-8.82	8.77	8.50-9.05
	55-64	18.76	18.20-19.34	19.24	18.66-19.85
	65+	69.65	67.63-71.72	71.38	69.27-73.55
Age x both intellectual disabilities and autism	0-15 (reference)	-	-	-	-
	16-24	-	-	0.63	0.51-0.79
	25-34	-	-	0.74	0.57-0.95
	35-44	-	-	0.39	0.30-0.52
	45-54	-	-	0.29	0.22-0.38
	55-64	-	-	0.22	0.16-0.29
	65+	-	-	0.22	0.17-0.30
Constant	-	0.01	-	0.01	-

For peer review only - http://bmjopen.bmj.com/site/about/guidelines.xhtml

Table 6: Independent predictors of physical disability in the whole population

Characteristic		Regression 1		Regression 2 (including the interaction term: age x co-occurring intellectual disabilities and autism)	
		Odds ratio	95% confidence interval	Odds ratio	95% confidence interval
Co-occurring intellectual disabilities and autism	Not present (reference)	-	-	-	-
	Co-occurring intellectual disabilities and autism	61.16	57.60-64.94	157.54	144.58-171.66
Gender	Male (reference)	-	-	-	-
	Female	1.06	1.06-1.07	1.06	1.06-1.07
Sex	0-15 (reference)	-	-	-	-
	16-24	1.44	1.39-1.50	1.57	1.51-1.64
	25-34	2.60	2.52-2.69	2.86	2.76-2.96
	35-44	5.87	5.70-6.04	6.47	6.27-6.67
	45-54	10.61	10.31-10.91	11.66	11.32-12.02
	55-64	20.73	20.17-21.31	22.76	22.10-23.44
	65+	43.68	42.52-44.88	47.89	46.53-49.30
Age x both intellectual disabilities and autism	0-15 (reference)	-	-	-	-
	16-24	-	-	0.41	0.36-0.47
	25-34	-	-	0.34	0.29-0.41
	35-44	-	-	0.12	0.10-0.15
	45-54	-	-	0.08	0.06-0.09
	55-64	-	-	0.04	0.03-0.06
	65+	-	-	0.05	0.040-0.07
Constant	-	0.01	-	0.01	-

For peer review only - http://bmjopen.bmj.com/site/about/guidelines.xhtml

STROBE Statement—Checklist of items that should be included in reports of ***cross-sectional studies***

	Item No	Recommendation
**Title and abstract**	1	(*a*) Indicate the study's design with a commonly used term in the title or the abstract<br>Page 1 (title)
		(*b*) Provide in the abstract an informative and balanced summary of what was done and what was found<br>Page 2
**Introduction**		
Background/rationale	2	Explain the scientific background and rationale for the investigation being reported<br>Pages 4-7
Objectives	3	State specific objectives, including any pre-specified hypotheses<br>Page 7, line 9
**Methods**		
Study design	4	Present key elements of study design early in the paper<br>Page 8-11
Setting	5	Describe the setting, locations, and relevant dates, including periods of recruitment, exposure, follow-up, and data collection<br>Page 7-8
Participants	6	(*a*) Give the eligibility criteria, and the sources and methods of selection of participants<br>Page 7-8
Variables	7	Clearly define all outcomes, exposures, predictors, potential confounders, and effect modifiers. Give diagnostic criteria, if applicable<br>Page 8-10
Data sources/ measurement	8*	For each variable of interest, give sources of data and details of methods of assessment (measurement). Describe comparability of assessment methods if there is more than one group<br>Page 7-10
Bias	9	Describe any efforts to address potential sources of bias<br>Page 9-10
Study size	10	Explain how the study size was arrived at<br>Page 7-8
Quantitative variables	11	Explain how quantitative variables were handled in the analyses. If applicable, describe which groupings were chosen and why<br>Page 10-11
Statistical methods	12	(*a*) Describe all statistical methods, including those used to control for confounding<br>Page 10-11
		(*b*) Describe any methods used to examine subgroups and interactions<br>Page 10-11
		(*c*) Explain how missing data were addressed<br>Page 10
		(*d*) If applicable, describe analytical methods taking account of sampling strategy<br>N/A
		(*e*) Describe any sensitivity analyses<br>N/A

Results

For peer review only - http://bmjopen.bmj.com/site/about/guidelines.xhtml

Participants	13*	(a) Report numbers of individuals at each stage of study—eg numbers potentially eligible, examined for eligibility, confirmed eligible, included in the study, completing follow-up, and analysed<br>Page 11-12
		(b) Give reasons for non-participation at each stage<br>N/A
		(c) Consider use of a flow diagram<br>N/A
Descriptive data	14*	(a) Give characteristics of study participants (eg demographic, clinical, social) and information on exposures and potential confounders<br>Page 11-12, Table 1
		(b) Indicate number of participants with missing data for each variable of interest<br>Page 10
Outcome data	15*	Report numbers of outcome events or summary measures<br>Page 13-15
Main results	16	(*a*) Give unadjusted estimates and, if applicable, confounder-adjusted estimates and their precision (eg, 95% confidence interval). Make clear which confounders were adjusted for and why they were included<br>Page 12-14, Tables 3-6
		(*b*) Report category boundaries when continuous variables were categorized<br>N/A
		(*c*) If relevant, consider translating estimates of relative risk into absolute risk for a meaningful time period<br>N/A
Other analyses	17	Report other analyses done—eg analyses of subgroups and interactions, and sensitivity analyses<br>Page 12-14
**Discussion**		
Key results	18	Summarise key results with reference to study objectives<br>Page 15-17
Limitations	19	Discuss limitations of the study, taking into account sources of potential bias or imprecision. Discuss both direction and magnitude of any potential bias<br>Page 18-20
Interpretation	20	Give a cautious overall interpretation of results considering objectives, limitations, multiplicity of analyses, results from similar studies, and other relevant evidence<br>Page 15-20
Generalisability	21	Discuss the generalisability (external validity) of the study results<br>Page 18
**Other information**		
Funding	22	Give the source of funding and the role of the funders for the present study and, if applicable, for the original study on which the present article is based<br>Page 25

*Give information separately for exposed and unexposed groups.

Note: An Explanation and Elaboration article discusses each checklist item and gives methodological background and published examples of transparent reporting. The STROBE checklist is best used in conjunction with this article (freely

For peer review only - http://bmjopen.bmj.com/site/about/guidelines.xhtml

available on the Web sites of PLoS Medicine at http://www.plosmedicine.org/, Annals of Internal Medicine at http://www.annals.org/, and Epidemiology at http://www.epidem.com/). Information on the STROBE Initiative is available at www.strobe-statement.org.

For peer review only

For peer review only - http://bmjopen.bmj.com/site/about/guidelines.xhtml
